# Integrin CD11b positively regulates TLR4-induced signalling pathways in dendritic cells but not in macrophages

**DOI:** 10.1038/ncomms4039

**Published:** 2014-01-15

**Authors:** Guang Sheng Ling, Jason Bennett, Kevin J. Woollard, Marta Szajna, Liliane Fossati-Jimack, Philip R. Taylor, Diane Scott, Guido Franzoso, H. Terence Cook, Marina Botto

**Affiliations:** 1 Centre for Complement and Inflammation Research, Department of Medicine, Imperial College, Hammersmith Campus, Du Cane Road, London W12 0NN, UK; 2 Centre for Cell Signalling and Inflammation, Department of Medicine, Imperial College, Hammersmith Campus, Du Cane Road, London W12 0NN, UK; 3 Renal and Vascular Inflammation Section, Division of Immunology and Inflammation, Department of Medicine, Imperial College, Hammersmith Campus, Du Cane Road, London W12 0NN, UK; 4 Cardiff Institute of Infection and Immunity, Cardiff University School of Medicine, Tenovus Building, Heath Park, Cardiff CF14 4XN, UK

## Abstract

Tuned and distinct responses of macrophages and dendritic cells to Toll-like receptor 4 (TLR4) activation induced by lipopolysaccharide (LPS) underpin the balance between innate and adaptive immunity. However, the molecule(s) that confer these cell-type-specific LPS-induced effects remain poorly understood. Here we report that the integrin α_M_ (CD11b) positively regulates LPS-induced signalling pathways selectively in myeloid dendritic cells but not in macrophages. In dendritic cells, which express lower levels of CD14 and TLR4 than macrophages, CD11b promotes MyD88-dependent and MyD88-independent signalling pathways. In particular, in dendritic cells CD11b facilitates LPS-induced TLR4 endocytosis and is required for the subsequent signalling in the endosomes. Consistent with this, CD11b deficiency dampens dendritic cell-mediated TLR4-triggered responses *in vivo* leading to impaired T-cell activation. Thus, by modulating the trafficking and signalling functions of TLR4 in a cell-type-specific manner CD11b fine tunes the balance between adaptive and innate immune responses initiated by LPS.

## Abstract (web summary)

The signalling pathways that confer differences in the responses of dendritic cells and macrophages to LPS remain poorly understood. Here, Ling *et al.* report that the integrin C11b is required for LPS-induced TLR4 trafficking and signalling in dendritic cells but not in macrophages.

## Graphical abstract

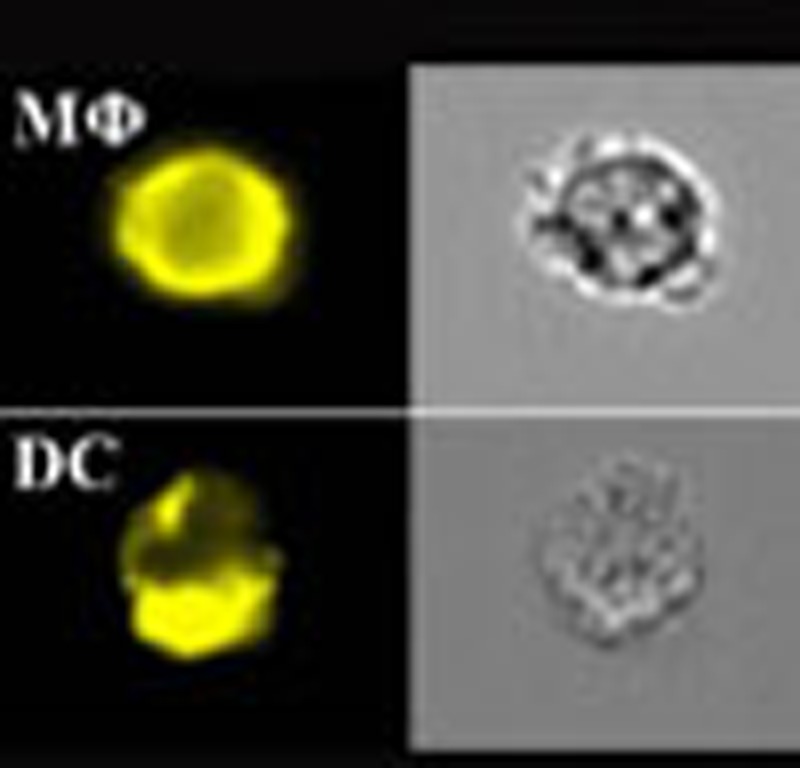

Lipopolysaccharide (LPS) is responsible for many of the pathogenic effects of Gram-negative bacteria and can also induce a protective adaptive immune response by acting as adjuvant. While diverse cell types can respond to LPS, they may mount qualitatively and quantitatively different responses^1^. The molecules and mechanisms dictating the cell type specificity of the LPS-induced effects remain poorly understood.

Dendritic cells (DCs) and macrophages (MΦs) arise from common myeloid precursors and share the ability to sample the tissue environment but have distinct effector functions^2^. Both types of cell sense microbes through pattern-recognition receptors, which initiate downstream signalling events^3^. However, as these cells mediate different immune functions, their LPS response must be tuned to reflect their roles.

Toll-like receptors (TLRs) are the best-characterized pattern-recognition receptors. TLR4 binds specifically to LPS and triggers two distinct sequential signalling pathways^3^,^4^. The first pathway (MyD88-dependent) is initiated from the plasma membrane, requires both MyD88 and TIRAP to activate NF-κB and initiate cytokine production^4^. TLR4 is then internalized into the endosome where a MyD88-independent pathway is triggered. These second signalling events are controlled by the adaptor molecules, TRAM (TRIF-related adaptor molecule) and TRIF (TIR-domain-containing adapter-inducing interferon-β)^5^, which activate Interferon (IFN) Regulatory Factor-3 (IRF3) leading to the subsequent production of type I IFNs and CCL5 (RANTES)^6^,^7^. Although LPS responses depend on a membrane-spanning complex formed by TLR4/MD-2, several molecules have been shown to act as co-receptors and/or accessory molecules and to regulate both positively and negatively LPS sensing/signalling^8^. One such regulator is CD11b.

CD11b, which pairs with CD18 to form a heterodimeric type 1 transmembrane receptor (CD11b/CD18; β_2_α_M,_) known as Mac-1/complement receptor 3 (CR3), has been suggested to contribute to the LPS signalling cluster^8^. CD11b is highly expressed on several cell types including MΦs and DCs and can bind to multiple ligands such as complement activation products (iC3b/C3b) and LPS^9^. CR3 activation is mediated by conformational changes often referred to as the ‘inside-out’ and ‘outside-in’ signalling pathways^10^.

CR3 plays a critical role in regulating inflammation and antimicrobial immunity^11^. *In vitro* and *in vivo* observations have also indicated a role for CD11b in TLR-triggered innate immune responses; however, the nature of this cross-talk remains controversial. On one hand, activation of CR3 through the binding of iC3b or fibrinogen delivers an ‘outside-in’ signal that leads to the downregulation of LPS-induced inflammation^11^,^12^. On the other hand, certain anti-CD11b antibodies or soluble mediators can act synergistically with LPS^13^,^14^. Furthermore, a recent study reported that CD11b could inhibit TLR signalling even in the absence of exogenous ligands^15^, whereas many other studies have shown that CD11b binds LPS and cooperates with TLR4 to elicit an optimal LPS response^16^,^17^,^18^,^19^.

In the present study, we demonstrate that CD11b can serve as a positive regulator of both TLR4-induced signalling pathways only on myeloid-derived DCs; however, it is dispensable in fully differentiated MΦs. The distinct role of CD11b in MΦs and DCs thereby provides an explanation for the apparently conflicting results in the literature and sheds new light on the regulation of the adaptive and innate immune responses triggered by LPS.

## Results

### CD11b does not affect TLR4-induced cytokine response in MΦs

**Figure 1:**
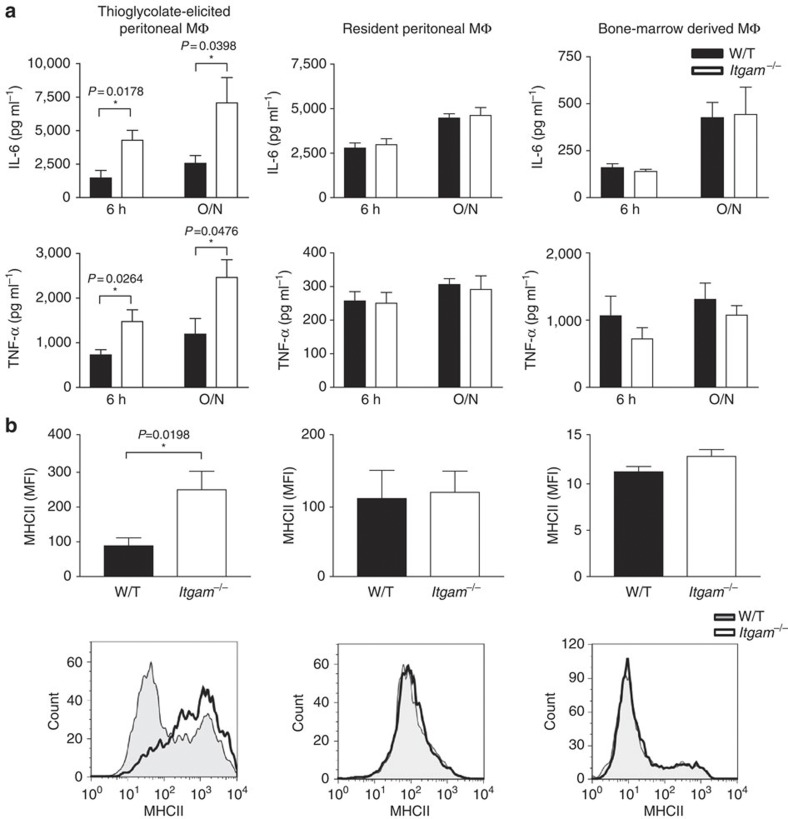
LPS-induced proinflammatory cytokine production by ΜΦs is not affected by CD11b deficiency. (**a**) Thioglycolate-elicited peritoneal ΜΦ, resident peritoneal ΜΦ and bone-marrow derived ΜΦs from W/T or *Itgam*^−*/*−^ mice were treated with LPS (10 ng ml^−1^) for 6 or 24 h and the production of IL-6 and TNF-α were measured using enzyme-linked immunosorbent assay (ELISA). (**b**) The surface expression of MHCII on different ΜΦs was examined using flow cytometry prior to the LPS stimulation. Data are expressed as delta MFI (MFI=test-isotype control). Data are represented as mean ±s.e.m. from two independent experiments with three mice per group. *P<0.05 (*t*-test).

There is growing interest in understanding how CD11b cross-regulates TLR4 signalling; however, the data in the literature are conflicting and thus the outcome of this cross-regulation remains unresolved^14^,^15^,^19^. TLR responses have been often investigated using thioglycolate-elicited peritoneal MΦs^15^. However, as the peritonitis induced by thioglycolate depends on complement activation^20^ and complement-deficient animals, including CD11b-deficient (*Itgam*^−/−^) mice, mount an abnormal cellular response^20^,^21^,^22^, we reasoned that resident peritoneal MΦs would provide a better controlled model. Consistent with a recent report^15^, we found that thioglycolate-elicited *Itgam*^−/−^ peritoneal MΦs produced more interleukin (IL)-6 and tumour necrosis factor alpha (TNFα) in response to LPS (Fig. 1a). However, these differences were detectable only with thioglycolate-elicited peritoneal MΦs, while resident peritoneal MΦs or bone marrow-derived MΦs (BM-MΦs) from *Itgam*^−/−^ and W/T animals secreted equivalent amounts of cytokines (Fig. 1a). Further analysis of the thioglycolate-elicited peritoneal MΦs showed that *Itgam*^−/−^ MΦs expressed markedly higher levels of major histocompatibility complex (MHC)II than their W/T counterparts, indicating a different state of activation. By contrast, MHCII expression on resident peritoneal MΦs and BM-MΦs was similar between W/T and *Itgam*^−/−^ mice (Fig. 1b). Additional analysis of the peritoneal lavage cells from thioglycolate-treated *Itgam*^−/−^ mice revealed not only that they express higher levels of MHCII, but also that the percentage of F4/80^hi^CD115^+^ cells was significantly higher (Supplementary Fig. S1). This is consistent with previous reports^21^,^22^ that CD11b deficiency affects the cellular composition of the peritoneal lavage following thioglycolate administration. Collectively, these data demonstrate that CD11b is not involved in the regulation of the LPS-triggered inflammatory response in steady-state MΦs.

### CD11b promotes MyD88-dependent TLR4 signalling in DCs

**Figure 2:**
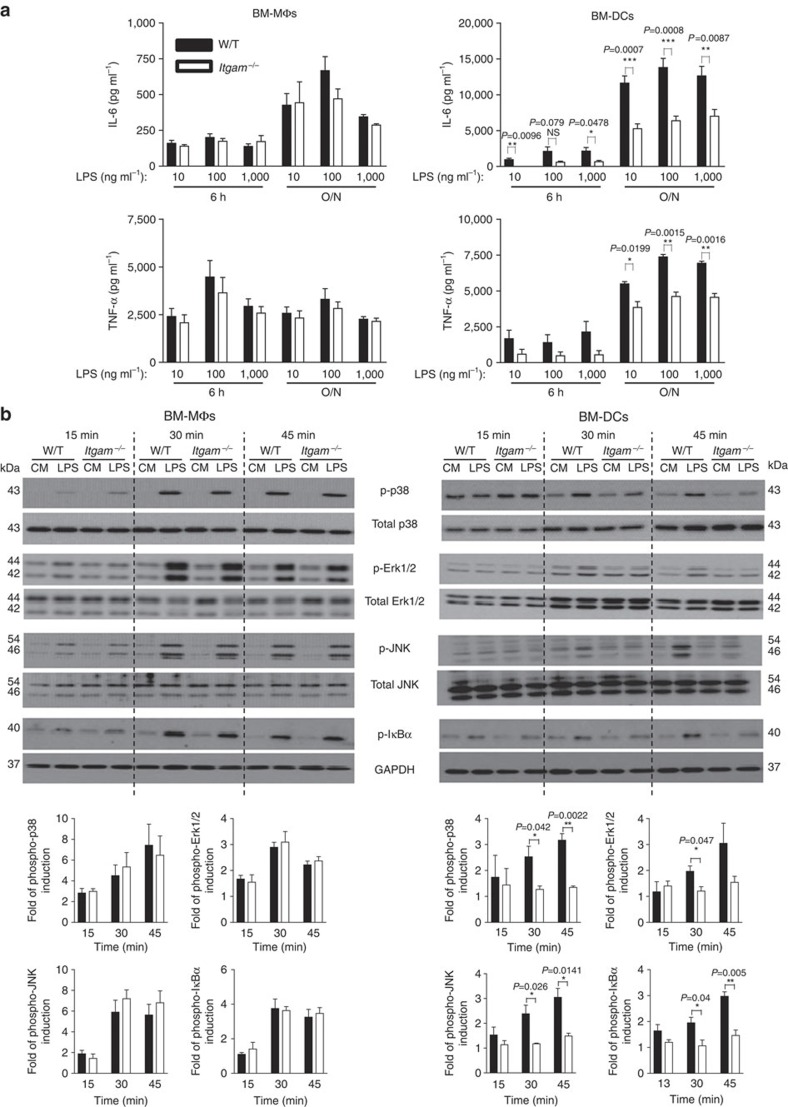
CD11b is required for MyD88-dependent TLR4 signalling and LPS-induced pro-inflammatory production in DCs. (**a**) BM-ΜΦs and BM-DCs from W/T or *Itgam*^−*/*−^ mice were treated with the doses of smooth LPS as indicated, and the production of IL-6 and TNF-α was measured at 6 h and overnight (O/N). Data are mean±s.e.m. from three independent experiments, three mice per group. (**b**) W/T or *Itgam*^−*/*−^ BM-ΜΦs and BM-DCs were left untreated (CM) or treated with 1 μg ml^−1^ of LPS for the time indicated. Phosphorylation of p38, Erk1/2, JNK and IκBα was examined using western blot. Quantification of band intensity showing the fold increase in phospho-protein levels relative to untreated cells and normalized to total protein levels. Data are mean±s.e.m. from three independent experiments. *P<0.05, **P<0.01, ***P<0.005 (*t*-test).

**Figure 3:**
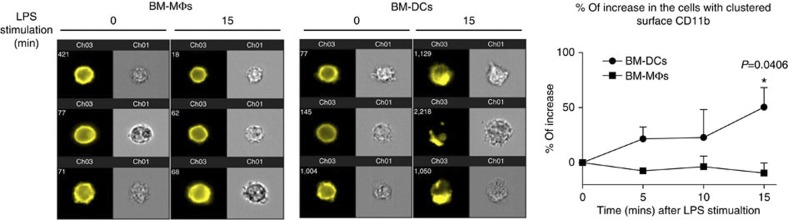
LPS stimulation induces surface CD11b clustering in DCs but not in ΜΦs. W/T BM-ΜΦs and BM-DCs were stimulated with 1 μg ml^−1^ of LPS for the times indicated. The percentage increase in the cells showing CD11b clustering was quantified using the ImageStream system software as described in the Methods section. Data represent mean±s.e.m. from three independent experiments. *P<0.05 (*t*-test).

We next compared the response of bone marrow-derived DCs (BM-DCs) and BM-MΦs over a range of LPS concentrations. We found that *Itgam*^−/−^ BM-DCs consistently secreted less IL-6 and TNFα, while *Itgam*^−/−^ BM-MΦs did not display any impaired production (Fig. 2a). Consistent with these data, the intensity of TLR4-induced MyD88-dependent phosphorylation of p38, Erk1/2, JNK and IkBα was markedly reduced in LPS-stimulated *Itgam*^−/−^ BM-DCs but not in *Itgam*^−/−^ BM-MΦs compared with the corresponding W/T cells (Fig. 2b and Supplementary Fig. S8). Similar cytokine results were observed with both adherent and floating bone marrow-derived myeloid cells differentiated *in vitro* with granulocyte–macrophage colony stimulating factor (GM-CSF) only (Supplementary Fig. S2b). These cells are variably defined in the literature as MΦs or DCs^23^,^24^,^25^, which may explain some of the conflicting results. Notably, irrespective of the culture conditions applied, *Itgam*^−/−^ and W/T myeloid cells displayed similar maturation status before LPS stimulation (Supplementary Fig. S2a). Moreover, unstimulated *Itgam*^−/−^ and W/T cells expressed similar levels of TLR4 and CD14 (Supplementary Fig. S2a). The presence of GM-CSF in the culture medium is critical for BM-DC differentiation, and thus the different role exhibited by CD11b on BM-DCs and BM-MΦs could be the result of GM-CSF-mediated cell programming rather than a lineage/differentiation-specific effect, as shown for other molecules^26^. To explore this possibility, we primed *Itgam*^−/−^ and W/T BM-MΦs with GM-CSF or GM-CSF plus IL-4 prior to LPS stimulation (Supplementary Fig. S3a). Under these conditions, the cells acquired some DC-like phenotypic features, yet we did not observe any difference in the cytokine production between W/T and *Itgam*^−*/*−^ cells indicating that the CD11b dependency in response to LPS is restricted to BM-DCs (Supplementary Fig. S3b,c). As BM-MΦs and BM-DCs exhibit different surface levels of TLR4 and CD14 (Supplementary Fig. S2c) and CR3 has been shown to bind LPS, we hypothesized that on cells bearing lower levels of TLR4 and CD14, such as BM-DCs, CD11b is required to promote the formation of the LPS recognition/signalling complex^18^,^19^,^27^. We therefore examined CD11b surface distribution after LPS stimulation using multispectral imaging flow cytometry. This analysis showed an increase in the percentage of BM-DCs displaying clustered CD11b after LPS stimulation. In contrast, the surface expression of CD11b remained evenly distributed in LPS-treated BM-MΦs (Fig. 3). In addition, using an antibody that loses affinity for TLR4 when the receptor interacts with LPS (MTS510)^28^, we found that, upon LPS stimulation, TLR4 staining on *Itgam*^−*/*−^ BM-DCs remained largely unchanged, while it was significantly decreased on W/T cells (Supplementary Fig. S4a). Collectively, these data demonstrate that CD11b facilitates LPS binding to TLR4 and acts as an additional signalling partner in the LPS receptor signalosome complex on BM-DCs, which express low levels of TLR4 and CD14, but not on MΦs. Thus CD11b contributes to the TLR4 signalling from the plasma membrane in a cell-type-specific manner.

### CD11b facilitates TLR4 endocytosis in BM-DCs

**Figure 4:**
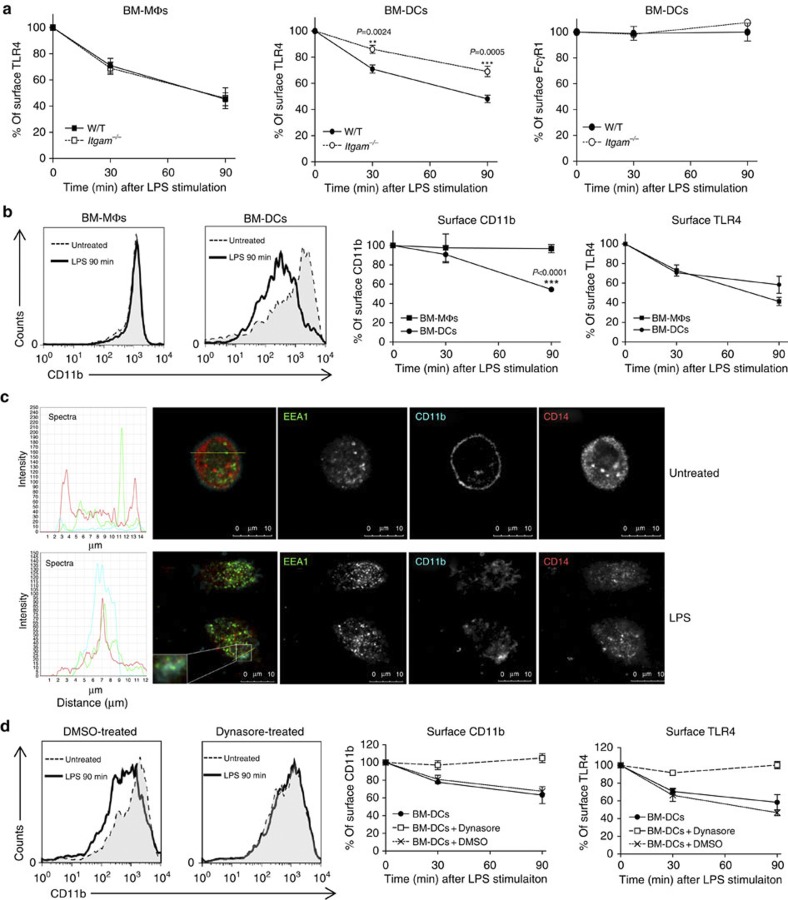
LPS induces CD11b endocytosis in DCs. (**a**) W/T or *Itgam*^−*/*−^ BM-ΜΦs and BM-DCs were treated with LPS (1 μg ml^−1^) for the times indicated. Receptor endocytosis was monitored with flow cytometry using the Sa15-21 mAb. Displayed are the MFIs of specific receptor staining (TLR4 or FcγR1) at each time point after LPS stimulation (MFI at time 0 as 100%). Data are representative of three independent experiments (mean±s.e.m., *n=3*). (**b**) W/T BM-ΜΦs and BM-DCs were treated with LPS (1 μg ml^−1^), and surface levels of TLR4 and CD11b were assessed using flow cytometry at the times indicated. Shown are the MFIs of specific receptor staining at each time point (MFI at time 0 as 100%). Data are representative of three independent experiments (mean±s.e.m., *n=3*). (**c**) W/T BM-DCs were left untreated (upper panel) or were treated with 1 μg ml^−1^ of LPS (lower panel) for 30 min. Cells were fixed, permeabilized and labelled with EEA1-alexa488 (green), CD11b-V450 (cyan) and CD14-alexa555 (red) antibodies, and analysed using confocal microscopy. Images are representative of at least two independent experiments. Scale bars=10 μm. Left of the merged image plotted the florescence spectra of each channel in the region of interest (ROI; yellow line). (**d**) W/T BM-DCs left untreated (circle) or treated with Dynasore (square) or dimethylsulphoxide (cross) were stimulated with LPS (1 μg ml^−1^). Surface levels of TLR4 and CD11b were measured at the times indicated. Displayed are the MFIs of specific receptor staining at each time point (MFI at time 0 as 100%). Data are representative of three independent experiments (mean±s.e.m., *n=3*). *P<0.05, **P<0.01, ***P<0.005 (*t*-test).

Recent work has suggested that TLR4 can be delivered into endosomes, where TRIF-dependent signalling can occur^5^, and that CD14 is essential for this endocytic pathway triggered by LPS^3^. We next explored whether CD11b can contribute to this process. We used the loss of cell surface expression as readout for TLR4 endocytosis and the production of RANTES as a surrogate for TRIF-mediated IRF3 activation^3^,^6^,^7^. As shown with the TLR4 signalling from the plasma membrane, the lack of CD11b significantly affected LPS-induced TLR4 endocytosis only in BM-DCs, but not in BM-MΦs (Fig. 4a and Supplementary Fig. S4b). The delayed internalization was specific as FcγR1 expression was largely unaffected by LPS stimulation (Fig. 4a). Consistent with the hypothesis that CD11b is part of the LPS receptor complex regulating TLR4 entry into BM-DCs, upon LPS stimulation CD11b was internalized in W/T BM-DCs, but not in BM-MΦs (Fig. 4b). Likewise, LPS-induced CD14 internalization was not affected by the lack of CD11b in BM-MΦs, but was inhibited in *Itgam*^−/−^ BM-DCs, indicating that the LPS-induced endocytic pathway in BM-DCs requires both molecules (Supplementary Fig. S5a). This was further investigated using confocal microscopy that showed co-staining of CD11b and CD14 in BM-DCs only after LPS stimulation (Fig. 4c). Both molecules appeared around LPS-induced macropinosomes, which stained positive for the early endosomal marker EEA1, confirming the flow cytometry data. In contrast, the expression level of CD11c (integrin α_X_) remained unchanged after LPS stimulation, demonstrating that this molecule is not involved in TLR4 endocytosis (Supplementary Fig. S5b). To demonstrate that CD11b was internalized by endocytosis like TLR4, we used dynasore that blocks endocytosis^7^. Dynasore treatment prevented the internalization of both TLR4 and CD11b in BM-DCs (Fig. 4d), confirming that CD11b is part of the TLR4–endocytic cluster.

### CD11b is required for LPS-induced RANTES secretion by DCs

**Figure 5:**
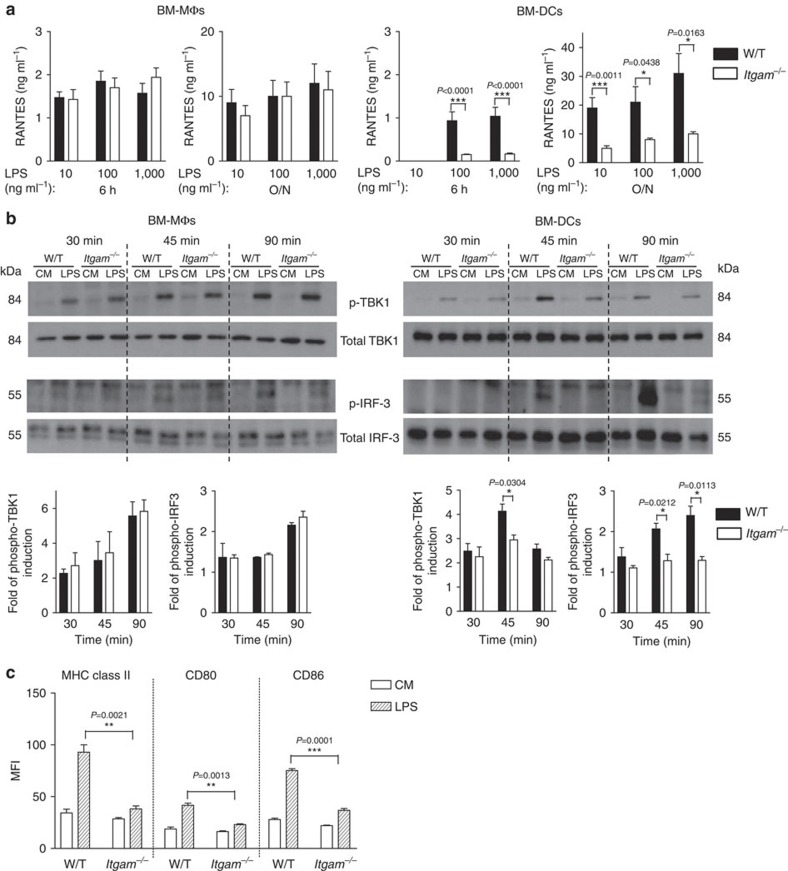
CD11b is required for TRIF-dependent TLR4 signalling in DCs. (**a**) W/T or *Itgam*^−*/*−^ BM-ΜΦs and BM-DCs were treated with the indicated doses of LPS and the amounts of RANTES secreted were measured using ELISA at 6 h and O/N. Data are mean±s.e.m. from three independent experiments with three mice per group. (**b**) W/T or *Itgam*^−*/*−^ BM-ΜΦs and BM-DCs were left untreated (CM) or treated with LPS (1 μg ml^−1^) for the time indicated. The presence of phosphorylated (p-)TBK-1 and IRF3 was assessed using western blot. Quantification of band intensity showing the fold increase in phospho-protein levels relative to untreated cells and normalized to total protein levels. Data are mean±s.e.m. from three independent experiments. (**c**) W/T or *Itgam*^−*/*−^ BM-DCs were left untreated (CM) or treated with 1 μg ml^−1^ of LPS for 24 h. DC maturation markers (MHC class II, CD80 and CD86) were assessed using flow cytometry. Data are expressed as delta MFI (MFI=test-isotype control). Data are representative of three independent experiments (mean±s.e.m., *n=3*) *P<0.05, **P<0.01, ***P<0.005 (*t*-test).

In agreement with the endocytic findings, *Itgam*^−/−^ BM-DCs, but not BM-MΦs, were markedly defective in LPS-induced TRIF-mediated RANTES production (Fig. 5a). Similar results were observed with non-adherent GM-CSF-differentiated BM-derived myeloid cells, which are widely used as BM-DCs (Supplementary Fig. S2b). Consistent with the cytokine data, TRIF-mediated TANK-binding kinase-I (TBK-1) and IRF3 phosphorylation were markedly diminished in LPS-treated *Itgam*^−/−^ BM-DCs compared with W/T BM-DCs, but similar in BM-MΦs (Fig. 5b and Supplementary Fig. S9). By contrast, no obvious differences were found in Akt or Syk phosphorylation in *Itgam*^−/−^ BM-DCs (Supplementary Fig. S5c and S11). Stimulation of BM-DCs with LPS induces upregulation of MHC class II and co-stimulatory molecules in a TRIF-dependent manner^29^. In keeping with the signalling data, *Itgam*^−/−^ BM-DCs displayed markedly diminished induction of MHCII, CD80 and CD86 after LPS stimulation (Fig. 5c) compared with the W/T BM-DCs. In addition, this defect was specific for BM-DCs, as MHCII expression was comparable in LPS-treated W/T and *Itgam*^−/−^ BM-MΦs (Supplementary Fig. S5d). Collectively, these data demonstrate that CD11b contributes to deliver TLR4 to endosomes and is required to induce TRIF-dependent signalling in BM-DCs.

**Figure 6:**
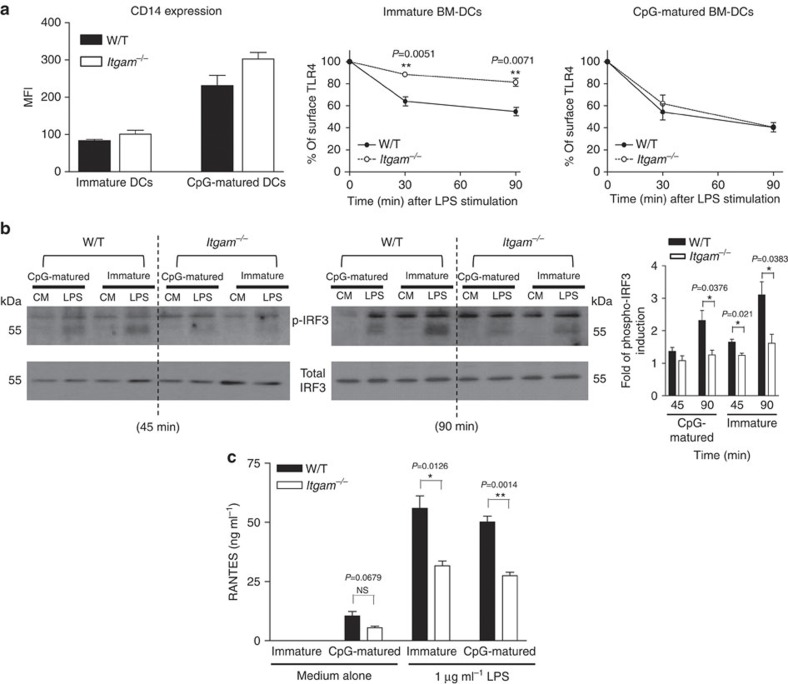
Upregulation of CD14 expression in *Itgam*^−*/*−^ BM-DCs matured with CpG DNA rectifies TLR4 endocytosis but not TRIF-dependent signalling events. BM-DCs were treated with 0.5 μM CpG DNA (CpG-matured) or left untreated (immature) for 18 h. (**a**) Surface expression of CD14 was assessed. Cells were then stimulated with 1 μg ml^−1^ of LPS and the surface levels of TLR4 were measured using flow cytometry with the Sa15-21 mAb at the times indicated. Displayed are the MFI of specific receptor staining at each time point (MFI at time 0 as 100%). (**b**) Immunoblot analysis of phosphorylated (p-)IRF3 in cell extracts from CpG-matured and -immatured DCs after stimulation with 1 μg ml^−1^ of LPS for 45 and 90 min. Quantification of band intensity showing the fold increase in p-IRF3 levels relative to untreated cells and normalized to total IRF3 levels. Data are mean±s.e.m. from three independent experiments. (**c**) The levels of RANTES secreted were measured using ELISA 24 h after LPS stimulation (1 μg ml^−1^). Data are representative of three independent experiments (mean±s.e.m., *n=3*). n.s=nonsignificant, *P<0.05, **P<0.01 (*t*-test).

Endosomal TLR4 endocytosis requires CD14 (ref. 3). If the restricted role of CD11b in BM-DCs is determined at least in part by the low expression of CD14, then increasing CD14 surface expression should restore TLR4 endocytosis and TRIF-dependent signalling. To test this, we increased the expression of CD14 by stimulating BM-DCs with CpG DNA, a TLR9 ligand that does not induce TRIF-IRF3 signalling events^3^,^30^. Consistent with previous data^3^, CpG DNA treatment enhanced surface levels of CD14 compared with immature BM-DCs. Interestingly, this increase was even more pronounced in *Itgam*^−/−^ BM-DCs (Fig. 6a). The CpG-matured *Itgam*^−/−^ BM-DCs, stimulated with LPS, internalized TLR4 similarly to CpG-matured W/T BM-DCs (Fig. 6a and Supplementary Fig. S10). However, the normalization in TLR4 endocytosis did not rectify the TRIF-dependent phosphorylation of IRF3 (Fig. 6b), nor did it restore RANTES production in *Itgam*^−/−^ BM-DCs to a level comparable to W/T BM-DCs (Fig. 6c). As TLR9 stimulation is known to involve MyD88/NF-κB signalling, IL-6 production was not assessed. Although with this approach we cannot rule out secondary effects of the CpG treatment, these data confirm that LPS-induced TLR4 endocytosis and signalling are independent events^3^ and suggest that CD11b on BM-DCs regulates TRIF/IRF3-dependent signalling from endosomes.

### T-cell response elicited by LPS-primed DCs depends on CD11b

TLR ligands such as LPS act as adjuvants for adaptive immune responses^29^ and TLR-induced pro-inflammatory cytokines, in particular IL-6 (ref. 31), regulate T-cell responses. To test whether the adjuvant effect of LPS required CD11b on DCs, we first examined whether the impaired cytokine response observed in LPS-treated *Itgam*^−/−^ BM-DCs could influence T-cell polarization *in vitro*. After 3-day stimulation with conditioned medium (CM) from LPS-stimulated DCs in the presence of transforming growth factor (TGF)-β, significantly more T regulatory cell and fewer Th17 cells were detected in the cultures from *Itgam*^−/−^ DCs compared with those from W/T DCs (Supplementary Fig. S6a).

**Figure 7:**
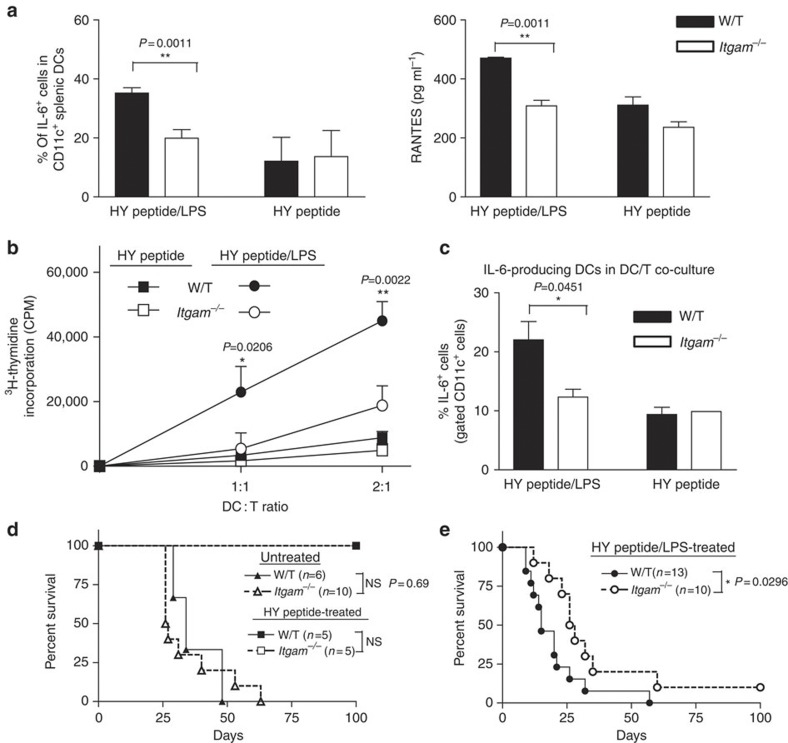
CD11b is required for the LPS adjuvant effect in DCs. (**a**–**c**) Female W/T or *Itgam*^−*/*−^ mice (*n*=3) were given i.n. twice 100 μg HY*Dby* peptide or peptide plus 3 μg LPS. Splenic CD11c^+^ cells isolated 24 h later were: (**a**) immediately stimulated with PMA/ionomycin for 6 h and the % of IL-6^+^ cells was determined using flow cytometry. The amount of secreted RANTES at 24 h was measured using ELISA; (**b**) cocultured with Marilyn T cells and the T-cell proliferation was assessed by ^3^H-thymidine uptake at 48 h. (**c**) Percentage of CD11c^+^ IL-6-producing cells in the 3-day DC/T cocultures was quantified by intracellular staining. Data are shown as mean±s.e.m., *n*=3, *P<0.05, **P<0.01 (*t*-test). (**d**) Survival of syngeneic male skin grafts transplanted on female W/T or *Itgam*^−*/*−^ mice that have been treated i.n. with HY^Db^*Uty* peptide (square symbols; *n*=5) or left untreated (triangle symbols; *n*=6–10, log-rank test). (**e**) Male skin graft survival after i.n. administration of HY peptide plus 3 μg LPS. Data shown are pooled results from two independent experiments, *P<0.05 (log-rank test).

We then used the male-specific Y-chromosome-encoded minor histocompatibility antigens (HY)^32^,^33^ model to assess the impact *in vivo*. Intranasal (i.n.) administration of the MHC class II-restricted HY peptide (HY^Ab^D*by*) to female mice can induce tolerance to HY-disparate skin grafts. However, this DC-mediated tolerance induction can be converted to immunization if LPS is co-administrated with the peptide^32^. We therefore treated W/T and *Itgam*^−/−^ female mice with HY peptide alone or HY peptide plus LPS (HY peptide/LPS) and isolated splenic DCs 24 h after the i.n. administration. In keeping with findings using BM-DCs, we found less IL-6 and RANTES producing splenic DCs in (HY peptide/LPS)-treated *Itgam*^−/−^ mice compared with (HY peptide/LPS)-treated controls and this difference was not observed in the groups receiving HY peptide alone (Fig. 7a). We then assessed the function of splenic DCs by incubating them with naive CD4^+^ T cells isolated from HY-TCR transgenic mice (Marilyn T cells)^34^. As previously published^33^, DCs isolated from peptide-treated W/T mice induced only a very weak proliferative response of the Marilyn T cells, while the i.n. inoculation of the peptide with LPS boosted the ability of DCs to induce T-cell proliferation (Fig. 7b). *Itgam*^−/−^ splenic DCs from the (HY peptide/LPS)-treated group, however, were severely impaired in their ability to elicit proliferation of antigen-specific T cells (Fig. 7b and Supplementary Fig. S6b). In addition, the percentage of IL-6^+^ DCs was reduced in the cocultures with *in vivo* (HY peptide/LPS)-primed *Itgam*^−/−^ DCs compared with the W/T counterparts (Fig. 7c). By contrast, no differences were detected between W/T and *Itgam*^−/−^ DCs that had been primed with the peptide only (Fig. 7c).

To test the impact of the impaired TLR4 signalling in *Itgam*^−/−^ BM-DCs *in vivo*, we investigated whether the absence of CD11b influenced the rejection pattern of syngeneic male skin graft on female recipients receiving the HY peptide plus LPS. First, we demonstrated that male skin grafted on untreated *Itgam*^−/−^ females was not rejected faster than on W/T females (Fig. 7d) and that i.n. administration of HY peptide prolonged equally the survival of syngeneic male skin grafts (Fig. 7d). We then assessed the male graft survival after i.n. administration of HY peptide/LPS. As expected, the rejection of the male skin graft in HY peptide/LPS-pretreated W/T female mice followed a faster time course (mean survival time (MST): 15 days) than that of male skin grafts in untreated W/T females (MST: 34 days). More importantly, HY peptide/LPS-pretreated W/T female mice rejected the male skin grafts significantly faster than the *Itgam*^−/−^ female mice with a MST of 15 and 27 days, respectively (Fig. 7e). In addition, after re-exposure to male antigen, a reduced expansion of Uty^+^CD8^+^ T cells and CD4^+^CD25^+^Foxp3^−^ T cells was detected in HY peptide/LPS-pretreated *Itgam*^−/−^ mice compared with their W/T counterparts, suggesting poor T-cell priming (Supplementary Fig. S6c). Taken together, these data confirmed *in vivo* the key modulating function of CD11b on LPS-primed DCs.

## Discussion

The recognition of microbial products by TLRs can trigger innate immune responses and prime adaptive immunity. Key to a fine-tuned response is the existence of distinct responses particularly in MΦs and DCs^1^. Therefore, there is a lot of interest in understanding how TLR activation is regulated in a cell-type-specific manner. Our discovery that CD11b controls LPS-induced signalling pathways in DCs but not in MΦs reconciles some of the conflicting reports in the literature and provides the molecular basis for distinct cellular responses to microbial products.

CD11b is usually viewed as a MΦ-specific marker but it is highly expressed on myeloid DCs. Here we demonstrate that CD11b is a key mediator of the adjuvant effect of LPS in DCs, but not in MΦs. In the absence of CD11b, DCs showed defective LPS binding to TLR4, impaired TLR4 signalling and poor induction of T-cell responses. LPS stimulation induces CD11b clustering only in DCs that express lower levels of TLR4 and CD14 compared with MΦs and thus require additional accessory molecules to act as co-receptors for the full TLR4 signalling transduction^16^,^18^,^35^,^36^. Our study demonstrates that CD11b promotes LPS binding to TLR4 and is involved in both TLR4 signalling pathways and that the CD11b dependency is not the result of a GM-CSF-mediated cell-programming effect. In this context, it is of note that CD11b-deficient GM-CSF bone marrow-derived adherent cells (Supplementary Fig. S2), which have a MΦ and DC overlapping gene expression profile^23^, secreted less IL-6 (MyD88-dependent) but similar amounts of RANTES (TRIF-dependent) compared with the W/T cells. The responses displayed by these cells are consistent with the findings reported by Kagan *et al.*^4^ They demonstrated that CD11b promoted TIRAP enrichment at the plasma membrane and had a positive regulatory effect restricted to the MyD88-dependent pathway^4^. On the basis of our observations, we would argue that these CD11b-mediated effects are likely to be restricted to a subtype of *in vitro* differentiated myeloid cells or a stage of the MΦ/DC lineage differentiation process.

Another novel finding of our study is the involvement of CD11b in the DC endosomal TRIF-dependent pathway. This pathway requires two steps: the initial TLR4 endocytosis and the subsequent TRIF-mediated signalling in the endosomes^7^. CD11b contributed to both stages. While CD14 has been shown to be essential for the delivery of TLR4 to the endosomal signalling machinery in DCs and MΦs^3^, we found that the lack of CD11b delayed TLR4 internalization only in DCs. Consistent with this accessory role of CD11b, LPS-induced Syk^3^ and Akt^37^ activations were not affected. Notably, while enhancing CD14 surface expression with CpG DNA treatment could compensate the defect in TLR4 internalization, it was unable to compensate defects in the TRIF/IRF3 signalling pathway. These data suggest two separate roles of CD11b in DCs: one in promoting TLR4 endocytosis and one in TRIF-mediated signalling, the latter appears to require CD11b irrespectively of the trafficking events. Our data also highlight the importance of CD11b in DC-mediated TLR4 signalling *in vivo*, a finding that challenges the traditional view of this molecule as a primary ‘phagocytic receptor’. The impaired T-cell response induced by *Itgam*^−*/*−^ DCs upon LPS stimulation is consistent with the poor priming and milder experimental allergic encephalomyelitis developed by *Itgam*^−*/*−^ mice when immunized with myelin oligodendrocyte glycoprotein peptide plus complete Freund’s adjuvant that activates TLR4 (ref. 38). The adjuvant effect of CD11b on LPS-primed DCs provides a protective mechanism by which mature DCs retain the ability to sense and respond to microbial encounters by inducing T-cell differentiation.

The demonstration that CD11b influences the trafficking and signalling functions of TLR4 in BM-DCs but not in MΦs may also explain some of the discrepancies in the literature. A cell-type-specific role can reconcile the observation that CD11b-deficient cells have an impaired IL-6 production in response to LPS^4^ with the finding that a tail-less version of CD11b is unable to promote phagocytosis but is still capable of eliciting a NF-κB-mediated response to LPS^16^. Similarly, the explanation for the conflicting findings that have been attributed to CD11b in various disease models^15^,^38^,^39^,^40^ may lie in the predominant role played by the different innate or adaptive immune cells in these experimental conditions. Furthermore, a recent study suggested that CD11b, in the absence of an exogenous ligand, can serve as negative regulator of TLR-triggered inflammatory responses^15^. As it appears that most of the observations in this study were obtained using thioglycolate-elicited peritoneal MΦs, we would argue that the reported hyper-responsiveness of *Itgam*^−*/*−^ MΦs to TLR stimulation^15^ is restricted to thioglycolate-elicited peritoneal MΦs. These MΦs from *Itgam*^−*/*−^ mice display an abnormal state of activation prior to TLR4 stimulation that is consistent with previous observations^21^,^22^, and the knowledge that thioglycolate-induced peritonitis requires complement activation^20^. Our data, however, do not exclude the possibility that ligation of CD11b to fibrinogen or iC3b can modulate TLR4 signalling in MΦs. Wang *et al.*^12^ reported indirect inhibitor effects mediated by activated CR3 when ligated with fibrinogen and we have obtained similar results using iC3b-coated particles (Supplementary Fig. S7). Interestingly, we found that this negative effect on TLR-triggered inflammatory responses is restricted to MΦs (Supplementary Fig. S7), suggesting a dynamic cross-talk between TLRs and integrins whose outcome is, at least in part, dependent on cell lineage/differentiation. Collectively, our data and previous reports support the hypothesis of a ‘signal-switch’ model^41^ whereby at sites of an acute inflammation a strong CR3-mediated inhibitory signalling effect on MΦs may contribute to dampen the potential toxicity of the cytokines generated by TLR stimulation, while during chronic inflammation or in steady-state conditions, tonic CD11b signalling triggered by low-avidity receptor ligation has no detectable effects on TLR responses by MΦs. These dual roles of CD11b on MΦs according to the surrounding would limit the tissue damage during an acute infection without compromising tissue homoeostasis.

In summary, our work demonstrates that CD11b facilitates LPS binding to TLR4 and controls trafficking and signalling functions of TLR4 in a cell-type-specific manner by acting as an important molecule in the cross-talk between adaptive and innate immune responses. From an evolutionary perspective, these differential effects of CD11b in MΦs and DCs ensure a coordinated and fine-tuned cellular response appropriate for the environmental trigger.

## Methods

### Mice

C57BL/6 mice were purchased from Charles River UK. C57BL/6-CD11b^−/−^ mice (*Itgam*^–/–^)^21^ were from Jackson Laboratory (Bar Harbor). Rag2^−/−^ Marilyn mice (TCR transgenic for the HY^Ab^*Dby* peptide NAGFNSNRANSSRSS) were a kind gift from Dr O Lantz^34^. Two to three months old female mice were used. All animals were handled in accordance with the institutional guidelines and procedures approved by the UK Home Office in accordance with the Animals (Scientific Procedures) Act 1986.

### Cell culture

Thioglycolate-elicited MΦs were isolated on day 4 from the peritoneum of mice injected intraperitoneally with 0.5 ml of 3% thioglycolate. Resident peritoneal MΦs were recovered from naive mice. BM-MΦs and BM-DCs were generated by culturing bone marrow cells with 20 ng ml^−1^ rM-CSF (PeproTech) for 7 days and with 16.67 ng ml^−1^ rGM-CSF (PeproTech) plus rIL-4 (5 ng ml^−1^, PeproTech) for 6 days, respectively. Cells were stimulated with TLR4-grade LPS from *Escherichia Coli* O111:B4 (Alexis Biochemicals). To enhance surface CD14 expression, BM-DCs were stimulated with 0.5 μM GpG (ODN 1826, Alexis Biochemicals) for 18 h. Splenic DCs were isolated by CD11c MACs beads (Miltenyi Biotec) and Marilyn cells were purified with CD4^+^ T cells isolation kit II (Miltenyi Biotec).

### Flow cytometry

BM-MΦs and BM-DCs were stained with the following antibodies for phenotypic analysis: Fluorochrome-conjugated monoclonal antibodies against CD11b (M1/70; dilution 1:200), CD11c (N418; dilution 1:100), CD115 (AFS98; dilution 1:100), CD80 (16-10A1; dilution 1:100), CD86 (GL1; dilution 1:100), F4/80(BM8; dilution 1:50) and MHC class II (AF6-120.1; dilution 1:100) were from BD Biosciences Pharmingen. Monoclonal antibodies against TLR4 (Sa15-21 and MTS510) were from BioLegend. The Sa15-21 antibody was labelled with biotin (Sigma). Both anti-TLR4 antibodies were added at a dilution of 1:50. PerCp-Cy5.5-conjugated (dilution 1:50) and biotin-conjugated (dilution 1:100) monoclonal antibodies against CD14 (Sa2-8) were from eBioscience. Data were acquired using a FACSCalibur (Becton-Dickinson) and expressed as delta the mean fluorescent intensity (MFI of the sample−MFI of the isotype control).

### ImageStream data acquisition and analysis

Cells were stimulated with 1 μg ml^−1^ of LPS in polypropylene tube for different time points. After being fixed with 1% paraformaldehyde the cells were stained with PE-conjugated anti-mouse CD11b (dilution 1:100). Images of 10,000 cells were acquired for each sample on the ImageStream imaging cytometer System 100 (Amnis Corp). The data were analysed using the ImageStream Data Exploration and Analysis Software (IDEAS, Amnis). Receptor clustering was analysed as described^42^.

### Immunofluorescence microscopy

BM-DCs were grown on Bio-coat coverslips (BD biosciences), fixed in 4% paraformaldehyde, permeabilized with 0.1% saponin and blocked with 5% goat serum. Samples were incubated with anti-CD11b-V450 (BD Biosciences, dilution 1:50), anti-CD14-biotin (ebioscience; dilution 1:50) and rabbit anti-EEA1 (Cell Signaling Technology; dilution 1:100). Secondary antibodies Alexa Fluor 555-conjugated Streptavidin and Alexa Fluor 488-conjugated goat-anti-rabbit IgG were both from Molecular Probes and were added at a dilution of 1:100. Samples were then analysed with a Leica SP5 confocal microscope. Colocalization spectra of each channel in the region of interest were analysed using the Lecia LAS AF software.

### Immunoblotting

After LPS stimulation, cells were lysed with RIPA buffer (Sigma) in the presence of protease inhibitor cocktail (Sigma) and phosphatase inhibitor cocktail 2 (Sigma). Proteins were fractioned using 10 or 12% SDS–PAGE and analysed using immunoblotting with the following antibodies: antibody specific for p38 MAPK phosphorylated at Thr180/Tyr182 (3D7), Akt phosphorylated at Ser473, Erk1/2 phosphorylated at Thr202/Tyr204 (197G2), IRF3 phosphorylated at Ser396 (4D4G), IκBα phosphorylated at Ser32/36 (5A5), JNK phosphorylated at Thr183/185 (81E11), Syk phosphorylated at Tyr525/526 (C87C1), TBK-1 phosphorylated at Ser172 (D52C2), p38 MAPK, Akt, Erk1/2 (137F5), IRF3(D83B9), JNK, Syk, TBK-1 (D1B4) and GAPDH (14C10). All antibodies were from Cell Signaling Technology and were used at a dilution of 1:1,000. Densitometry analysis on the bands was performed using the NIH Image J software and normalizing the data to total protein levels.

### T-cell polarization assay

BM-DCs were stimulated with 1 μg ml^−1^ LPS, and CM was collected after 18 h. CD4^+^ T cells were purified from naive W/T mice and activated with plate-bound anti-CD3 (2 μg ml^−1^; Biolegend) and soluble anti-CD28 (5 μg ml^−1^; eBioscience) in the presence of recombinant TGF-β1 (5 ng ml^−1^, PeproTech). CM from W/T or *Itgam*^−/−^ DCs was added to the T-cell culture at a ratio of 1:1. On day 3, the cells were restimulated with 10 ng ml^−1^ of PMA (Sigma) and 250 ng ml^−1^ of Ionomycin (Calbiochem) in the presence of GolgiStop (BD Pharmingen) for 5 h. Cells were then intracellularly stained with PE-conjugated anti-IL-17 (TC11-18H10; dilution 1:100) and FITC-labelled anti-IFN-γ (XMG1.2; dilution 1:100) (BD Pharmingen). For the detection of T regulatory cells, cells were intracellularly stained with antigen-presenting cell-conjugated Foxp3 (FJK-16s; dilution 1:100) (eBioscience) before the PMA/Ionomycin re-stimulation.

### Antigen presentation assay and skin graft

Two doses of 100 μg HYA^b^*Dby* peptide with and without 3 μg LPS were administered intranasally to mice. Splenic DCs were isolated 1 day after. Marilyn cells were added to DCs at 1:1 or 2:1 ratio. T-cell proliferation was assessed by thymidine incorporation after 48 h. To assess IL-6-producing cells in the coculture, cells were restimulated with 10 ng ml^−1^ of PMA and 250 ng ml^−1^ of Ionomycin in the presence of GolgiStop for 5 h. Phosphate-buffered saline (20 μl) containing 100 μg HYA^b^*Dby* peptide with or without 3 μg LPS were administered intranasally on three consecutive days to W/T and *Itgam*^−*/*−^ female mice. Ten days later, the mice were grafted with syngeneic male and female skin on the lateral thorax^32^.

### Detection of HY-specific T cells in grafted mice

One hundred days after skin grafting, mice were boosted with 5 × 10^6^ male splenocytes intraperitoneally. Seven days later, spleen cells (2 × 10^6^) were cultured with irradiated male splenocytes (2 × 10^6^) and 10 IU ml^−1^ recombinant mouse IL-2 (PeproTech). Live cells were harvested on day 6, cells were stained with CD8a (FITC-labelled anti-mouse CD8a, 53–6.7; dilution 1:100) and HY^Db^*Uty* PE tetramer (ProImmune; 3 μl) to detect HY-specific CD8^+^ T cells. To determine the percentage of activated CD4^+^ T cells, cells were stained with CD4 (FITC-labelled anti-mouse CD4, L3T4), CD25 (PE-labelled anti-mouse CD25, PC61) and Foxp3 (APC-labelled anti-mouse Foxp3, FJK-16s); antibodies were added at 1:100 dilution.

### Statistical analysis

Comparisons between two groups were performed using two-tailed unpaired Student’s *t*-test or Mann–Whitney test. Graft survival was compared using the log-rank (Mantel–Cox) test. Statistical significance is defined as *P*<0.05.

## Author contributions

G.S.L. performed the experiments, interpreted the data and wrote the manuscript; J.B., K.J.W., M.S. and L.F.-J. performed the experiments; P.R.T. designed experiments and edited the manuscript; D.S. assisted with data interpretation; G.F. and H.T.C. discussed the data and edited the manuscript; M.B. conceived, designed the study and wrote the manuscript.

## Additional information

**How to cite this article:** Ling, G. S. *et al.* Integrin CD11b positively regulates TLR4-induced signalling pathways in dendritic cells but not in macrophages. *Nat. Commun.* 5:3039 doi: 10.1038/ncomms4039 (2014).

## Supplementary Material

Supplementary Information Supplementary Figures S1-S11
