# Supplementary material for: Integrin CD11b positively regulates TLR4-induced signalling pathways in dendritic cells but not in macrophages
Source: Nat Commun. 2014 Jan 15;5:3039. doi: 10.1038/ncomms4039 (PMC3905776; doi:10.1038/ncomms4039)
Supplement: Supplementary Information — Supplementary Figures S1-S11 [file ncomms4039-s1.pdf]

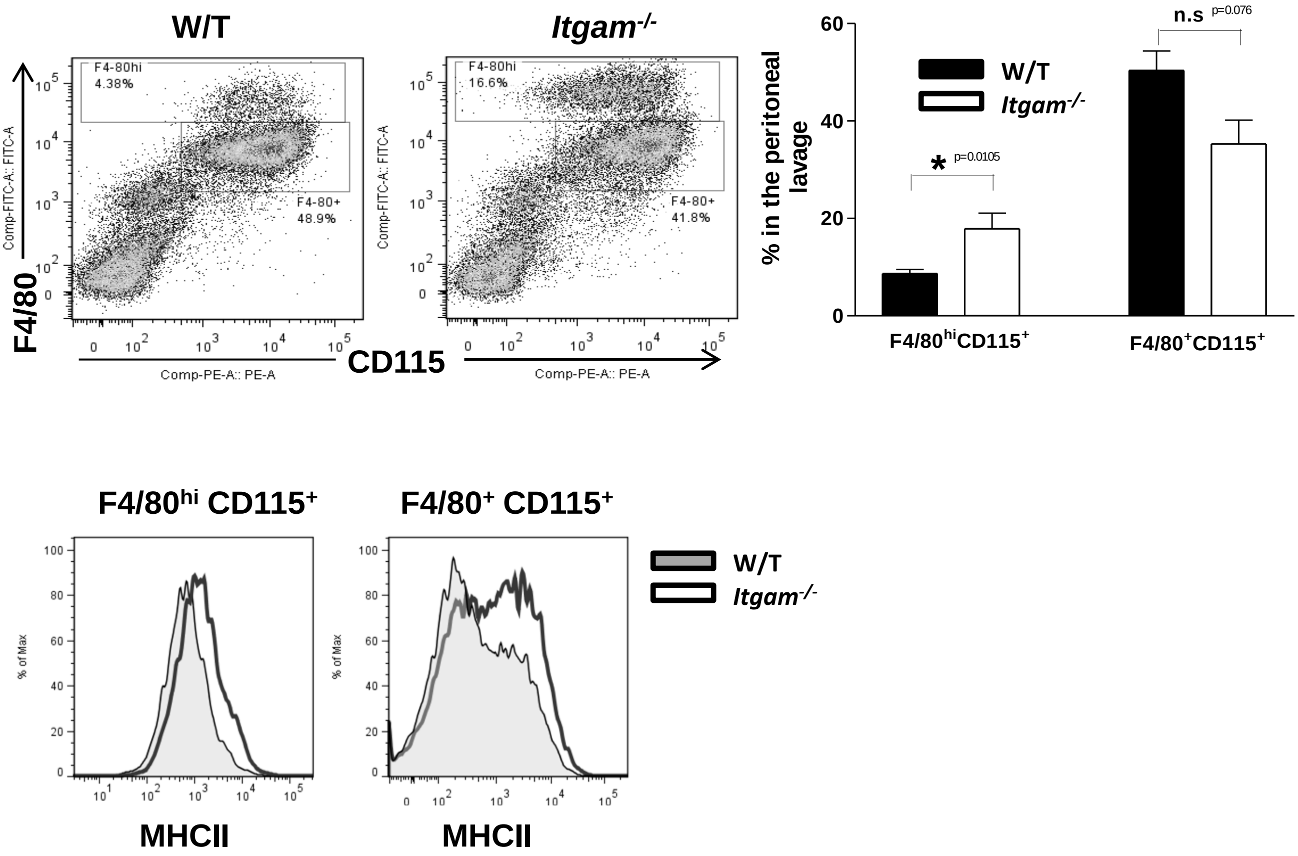

Supplementary Figure S1. CD11b deficiency affects the cellular responses to thigolycolate. Peritoneal cells were harvested from W/T or *Itgam*$^{-/-}$ mice 4 days after thioglycolate injection. Representative flow cytometry profiles of F4/80 and CD115 staining on peritoneal cells. Histogram showing the MHCII expression on different MΦ subpopulations (F4/80^{hi}/CD115^{+} and F4/80^{+}/CD115^{+}) and their respective percentage (mean ±S.E.M, n=3) in the peritoneal lavage from W/T and *Itgam*$^{-/-}$ mice. *$p<0.05$ (*t*-test).

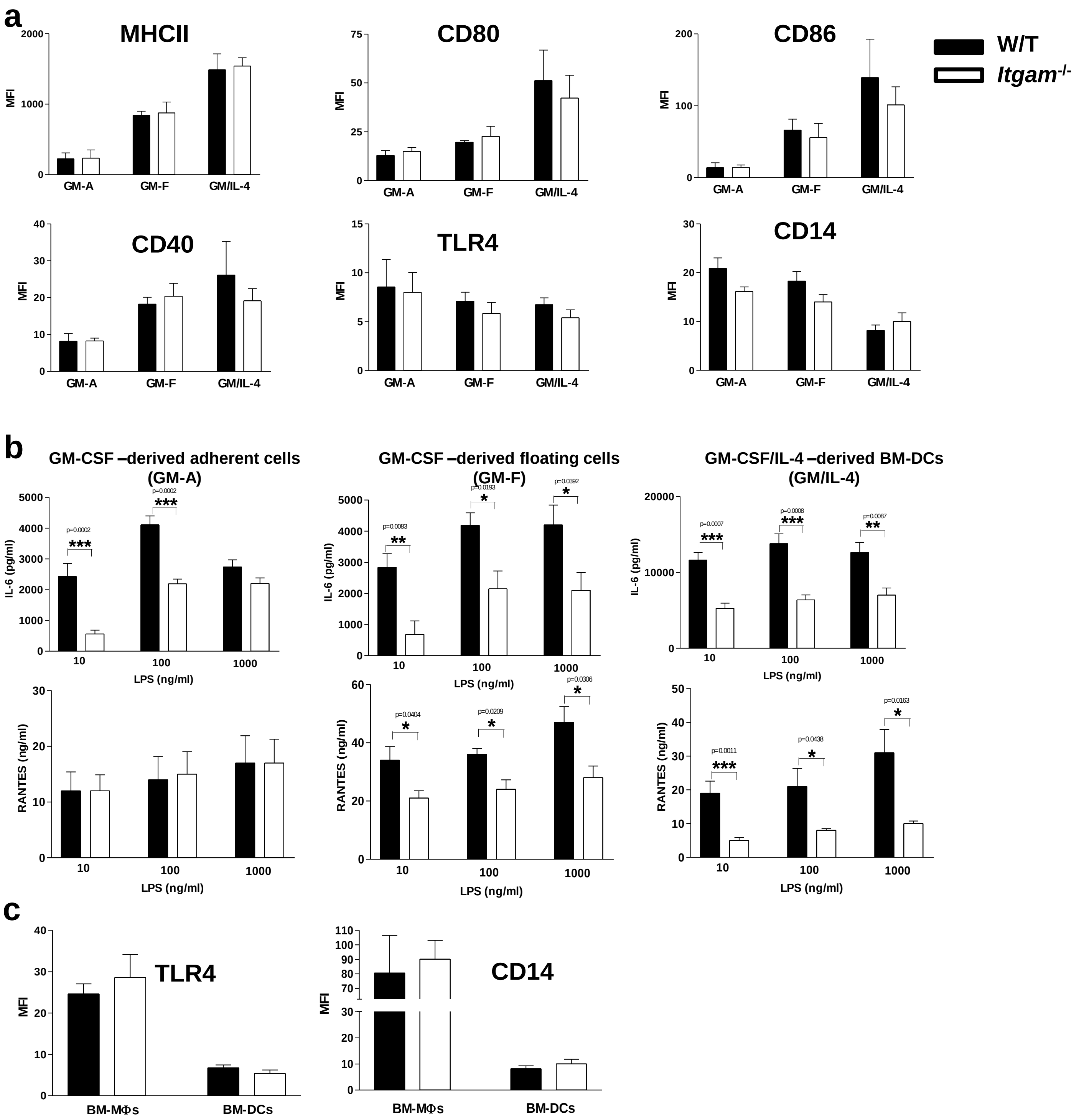

Supplementary Figure S2. CD11b is required for LPS-induced cytokine production by myeloid cells differentiated with GM-CSF alone. Bone marrow cells from W/T or *Itgam*$^{-/-}$ mice were cultured in 16.67ng/ml of GM-CSF. Floating cells (GM-F) and adherent cells (GM-A) were collected at day 6. BM-DCs (GM/IL-4) were also generated. **(a)** Surface expression of MHC class II, CD80, CD86, CD40, TLR4 and CD14 on unstimulated W/T and *Itgam*$^{-/-}$ BM-derived cells were examined by flow cytometry (MFI=test-isotype control) (mean ±S.E.M, *n=3*). **(b)** Cells were then treated with a range of LPS concentrations from 10ng/ml to 1000ng/ml. IL-6 and RANTES were measured by ELISA 24hrs later. Pooled data from three independent experiments are presented (mean ±S.E.M, n=9). **(c)** Surface expression of TLR4 and CD14 on unstimulated W/T and *Itgam*$^{-/-}$ BM-MΦs and BM-DCs measured by flow cytometry. Data are expressed as delta mean fluorescent intensity (MFI=test-isotype control) (mean ±S.E.M, *n=3*). **p<0.05, **p<0.01, ***p<0.005* (*t*-test).

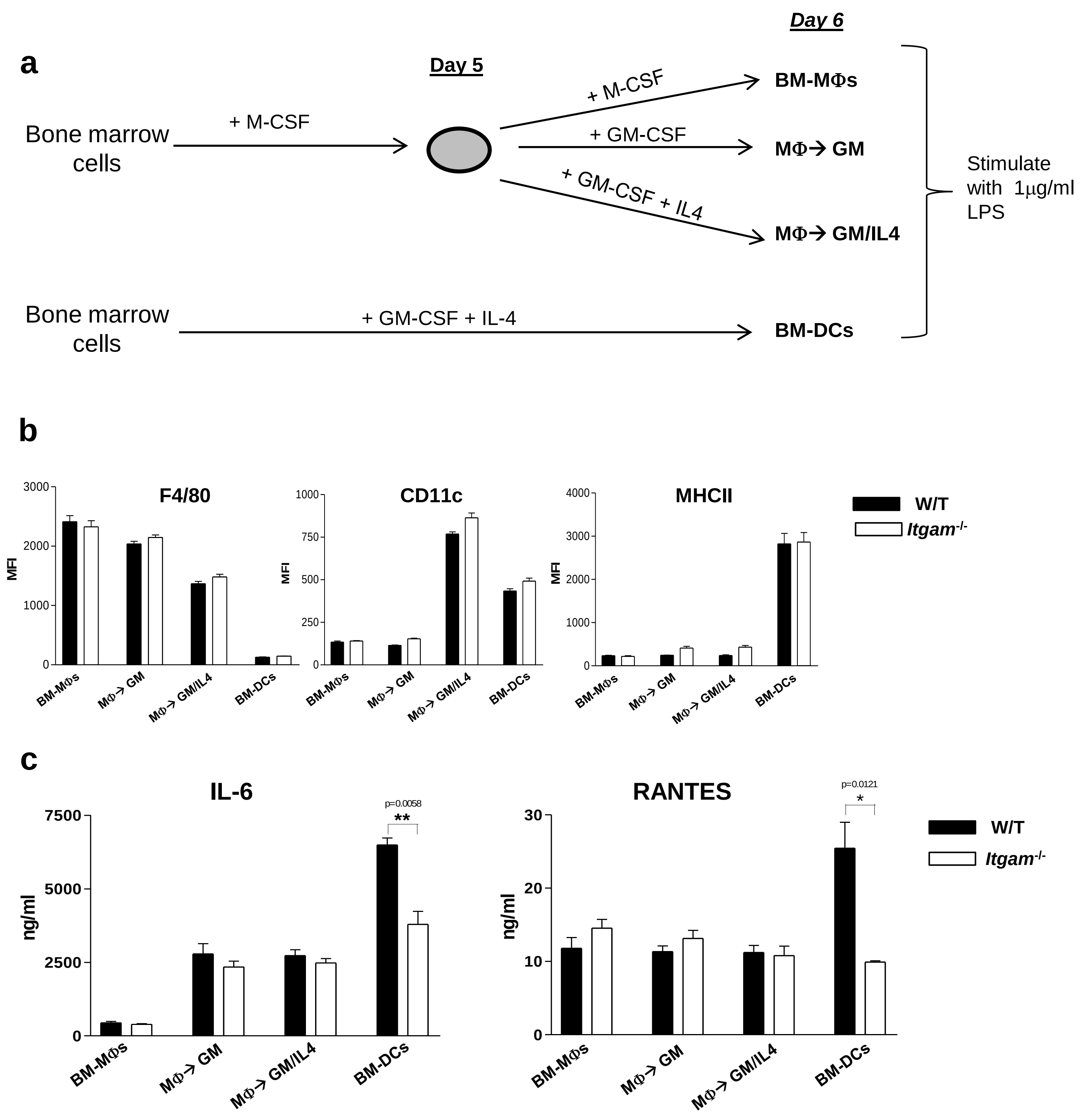

Supplementary Figure S3. CD11b dependency in LPS responses is restricted to BM-DCs and is a lineage/differentiation effect. (a) Schematic outline of the experimental design: bone marrow cells from W/T and *Itgam*$^{-/-}$ mice were differentiated with M-CSF for 5 days to obtain BM-MΦs. The M-CSF-containing medium was removed at day 5, the wells were washed with PBS and fed with media containing either GM-CSF or GM-CSF plus IL-4. BM cells conventionally differentiated into BM-DCs or BM-MΦs were used as controls. On day 6 cells were stimulated with 1μg/ml of LPS for 24hrs. **(b)** Surface expression of F4/80, CD11c and MHCII on unstimulated W/T and *Itgam*$^{-/-}$ BM-derived cells described in **(a)**. Data are expressed as delta mean fluorescent intensity (MFI=test-isotype control) (mean ±S.E.M, *n=3*). **(c)** Production of IL-6 and RANTES by the BM-derived cells described in **(a)** were measured by ELISA 24hrs after the LPS stimulation. Data represent mean ± S.E.M (n=3) $*p<0.05$, $**p<0.01$ (*t*-test).

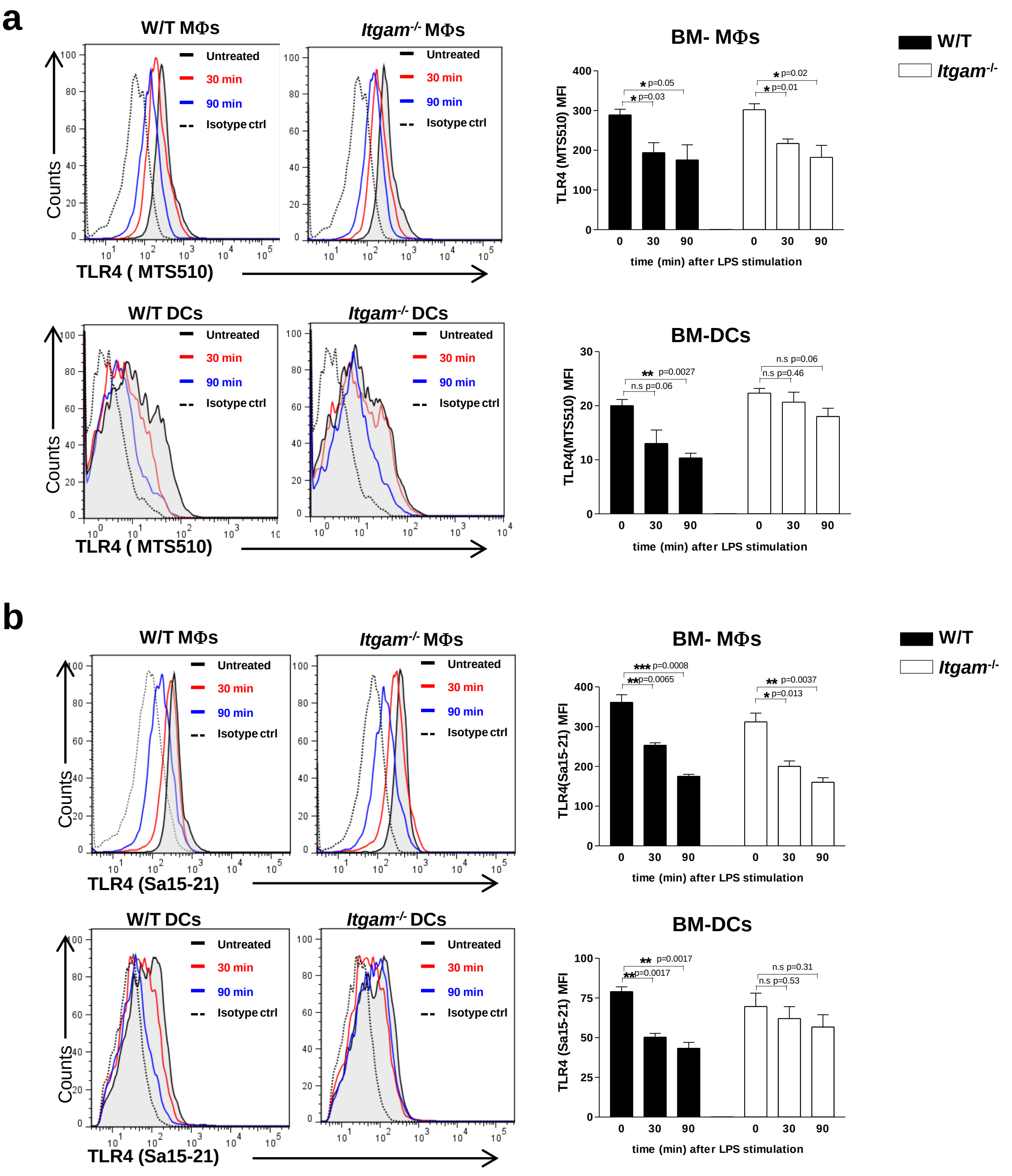

Supplementary Figure S4. CD11b is required for LPS-TLR4 binding and the subsequent TLR4 internalization in BM-DCs . W/T or *Itgam*$^{-/-}$ BM-MΦs and BM-DCs were treated with LPS (1μg/ml) for the times indicated. **(a)** Cell were stained with the MTS510 mAb, which recognizes only LPS-free TLR4-MD-2 complex, to measure LPS binding. **(b)** Cells were stained with the Sa15-21 mAb that detects the TLR4-MD-2 complex irrespective of the presence of LPS. The loss of surface Sa15-21 staining indicates receptor internalization. Representative histograms are shown. Data are expressed as delta mean fluorescent intensity (MFI = test – isotype control) (mean ±S.E.M, *n=3*). *$*p<0.05$, $**p<0.01$,$***p<0.005$* (*t*-test).

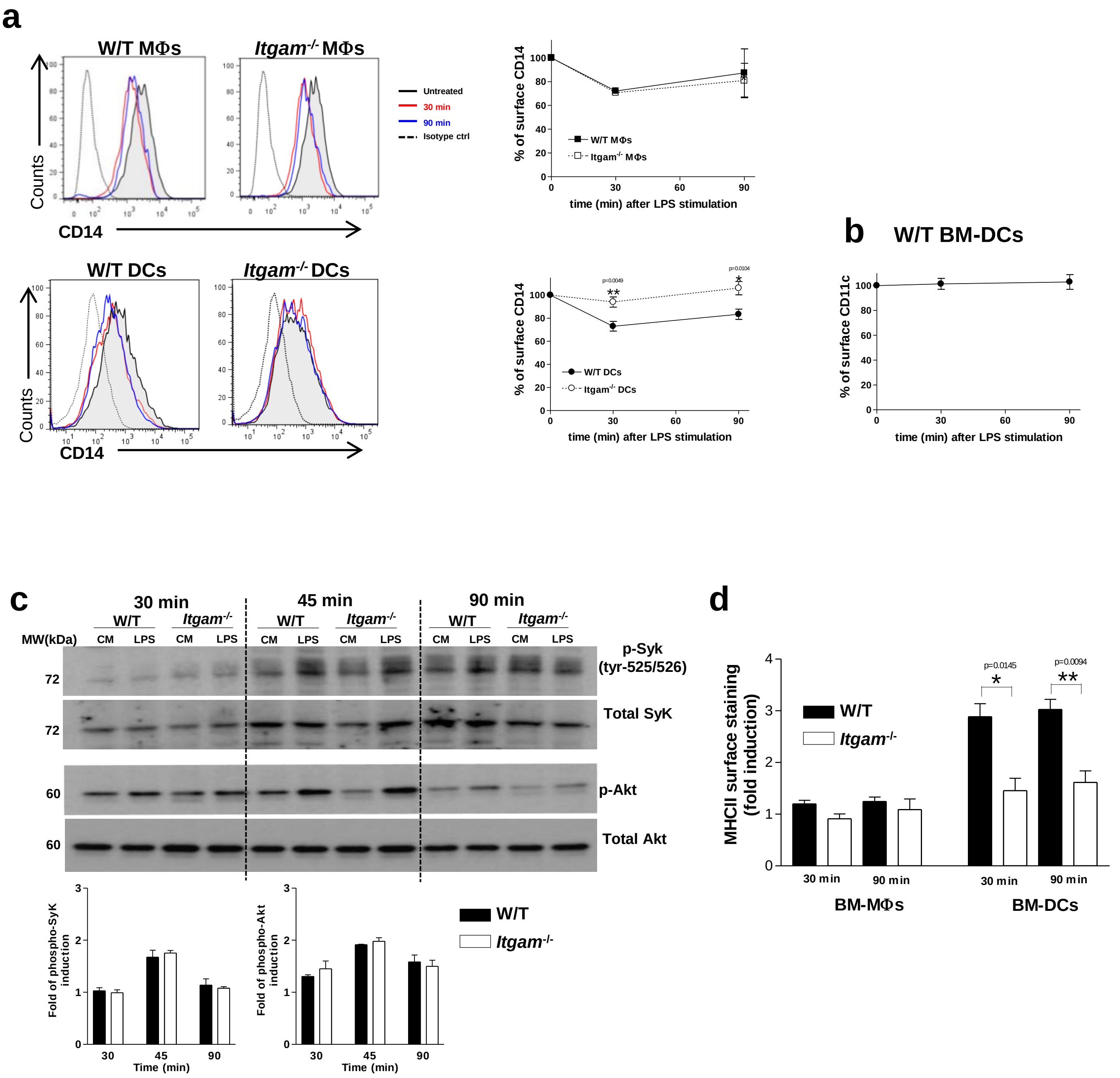

Supplementary Figure S5. CD11b contributes to CD14 internalization but not to Akt or Syk phosphorylation in BM-DCs. W/T or *Itgam*$^{-/-}$ BM-MΦs and BM-DCs were treated with LPS (1µg/ml) for the times indicated. Surface expression of **(a)** CD14 or **(b)** CD11c was measured by flow cytometry. **(a,b)** Displayed are the MFI of specific receptor staining at each time point (MFI at time 0 as 100%) (mean ±S.E.M, *n*=3). Representative histograms are shown. **(c)** W/T or *Itgam*$^{-/-}$ BM-DCs were left untreated (CM) or treated with LPS (1µg/ml) for the time indicated. The presence of phosphorylated (p-)Syk and Akt was examined by western blot. Quantification of band intensity showing the fold increase in phospho-protein levels relative to untreated cells and normalized to total protein levels (mean ±S.E.M, *n*=3). **(d)** W/T or *Itgam*$^{-/-}$ BM-MΦs and BM-DCs were treated with LPS (1µg/ml) for the times indicated. The fold increase in MHC class II expression was compared to untreated cells (mean ±S.E.M, *n*=3). $*p<0.05$, $**p<0.01$ (*t*-test).

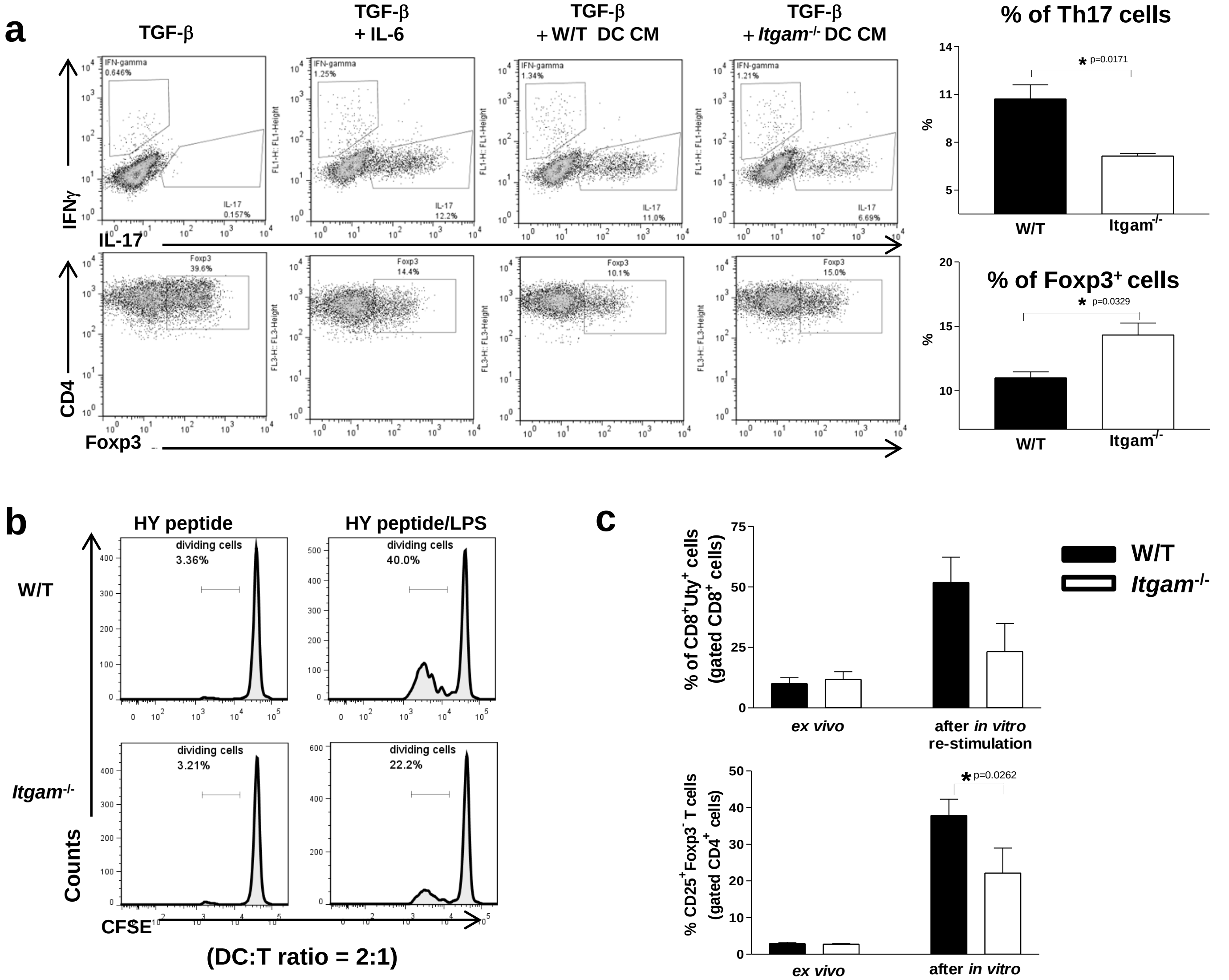

Supplementary Figure S6. CD11b regulates the T cell responses elicited by LPS-primed DCs *in vitro* and *in vivo*. **(a)** Naïve CD4$^+$T cells were stimulated with plate-bound anti-CD3/CD28 antibodies in the presence of TGF-β (with/without IL-6) or conditioned medium (CM) from LPS-treated W/T or *Itgam*$^{-/-}$ DCs. The percentage of Foxp3$^+$ cells was assessed at day 3. Cells were re-stimulated with PMA and ionomycin in the presence of GolgiSTOP for 5hrs and the percentage of Th17 cells was determined by intracellular staining. Data represent mean ±S.E.M from four independent experiments *(*p<0.05,t-test)*. **(b)** Female W/T or *Itgam*$^{-/-}$ mice (n=3) were given twice i.n. 100μg HY*Dby* peptide or peptide plus 3μg LPS. Splenic CD11c$^+$ cells, isolated 24hrs later, were co-cultured with Marilyn T cells for 72hrs. Representative flow cytometry histograms of CFSE-labelled Marilyn T cells. **(c)** HY peptide/LPS–treated W/T and *Itgam*$^{-/-}$ mice that had rejected the male grafts were boosted intraperitoneally with male spleen cells. The mice were sacrificed seven days later and the splenocytes were re-stimulated *in vitro* with irradiated male splenocytes for 1 week. The percentage of antigen specific CD8$^+$Uty$^+$ and CD4$^+$CD25$^+$Foxp3$^-$ T cells were analysed by flow cytometry before (*ex vivo*) and after the *in vitro* re-stimulation. Data represent mean ± S.E.M (n=7) *(*p<0.05 ,Mann-Whitney test)*.

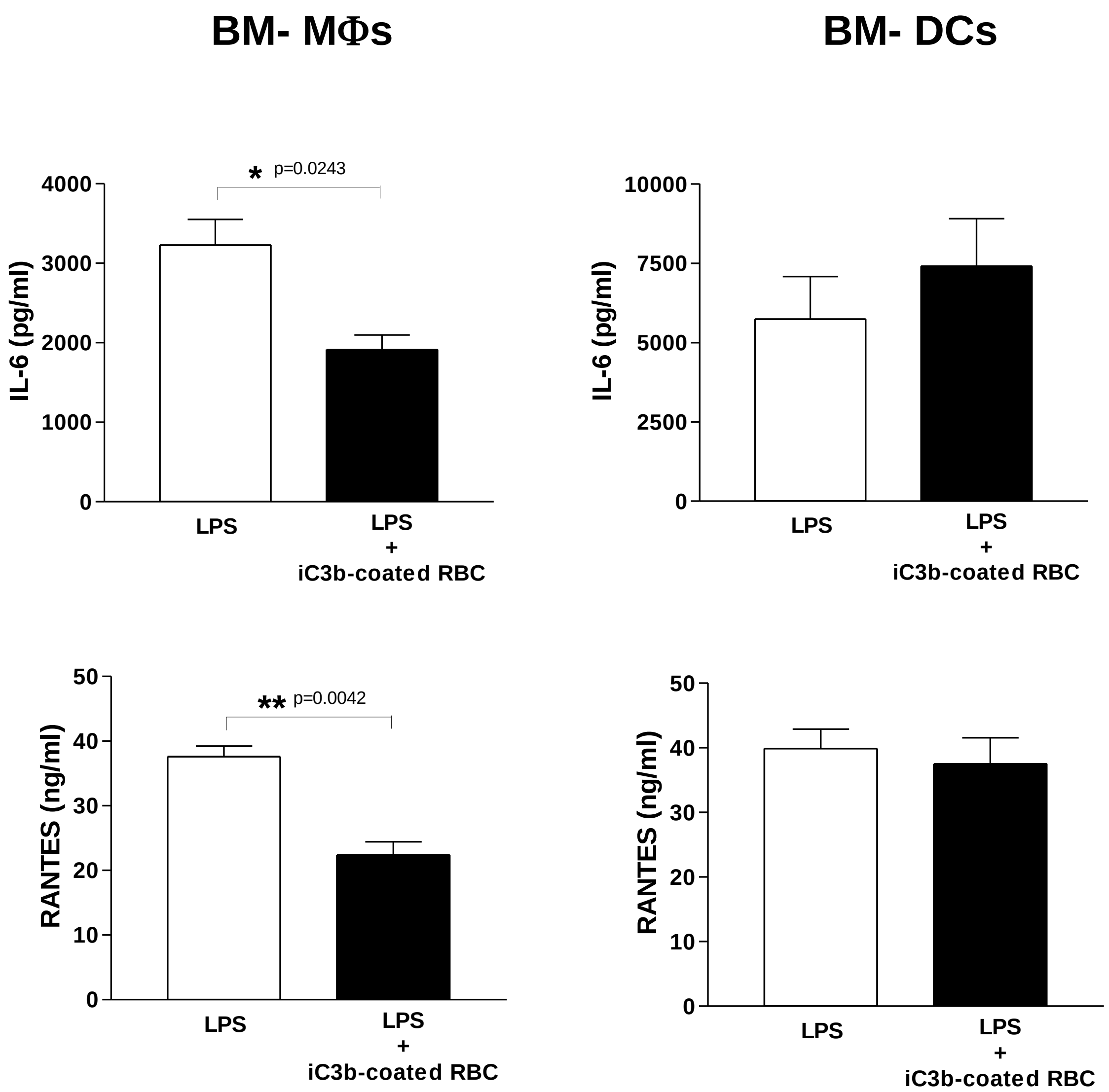

Supplementary Figure S7. iC3b-coated red blood cells (RBC) downregulate LPS-induced cytokine production in BM-MΦs but not BM-DCs. Guinea pig RBCs were opsonised with mouse C5-deficient serum to avoid complement lysis and were added to W/T BM-MΦs and BM-DCs at a 10:1 ratio (RBC:cells). Cells were then stimulated with 10ng/ml of LPS. The amounts of IL-6 and RANTES secreted were measured by ELISA at 24hr. Data represent mean ± S.E.M (n=3) **$p<0.05$, **$p<0.01$* (*t*-test).

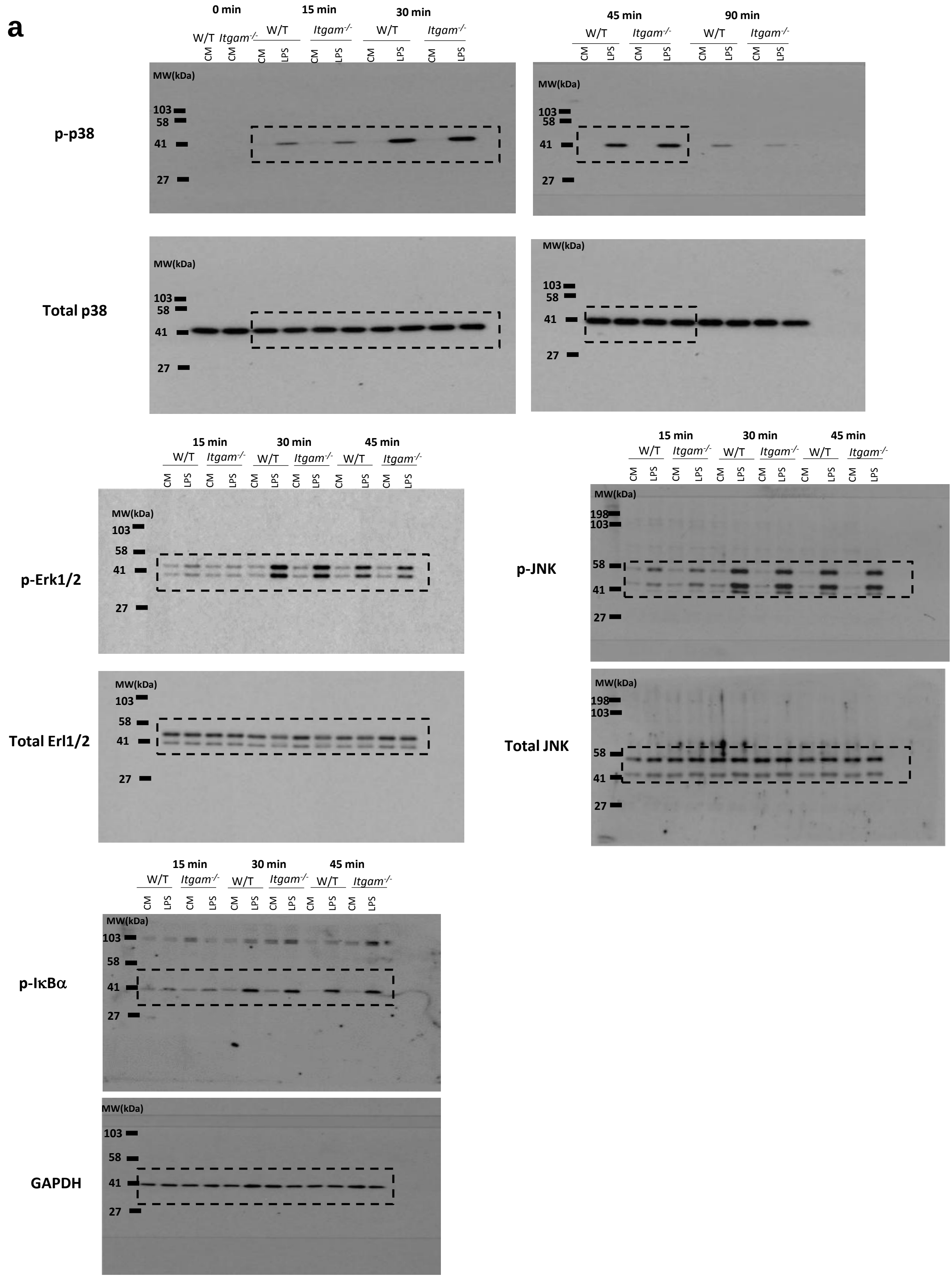
a
0 min
15 min
30 min
45 min
90 min
W/T
Itgam-/-
CM
LPS
MW(kDa)
198
103
58
41
27
p-p38
Total p38
p-Erk1/2
Total Erl1/2
p-JNK
Total JNK
p-IκBα
GAPDH

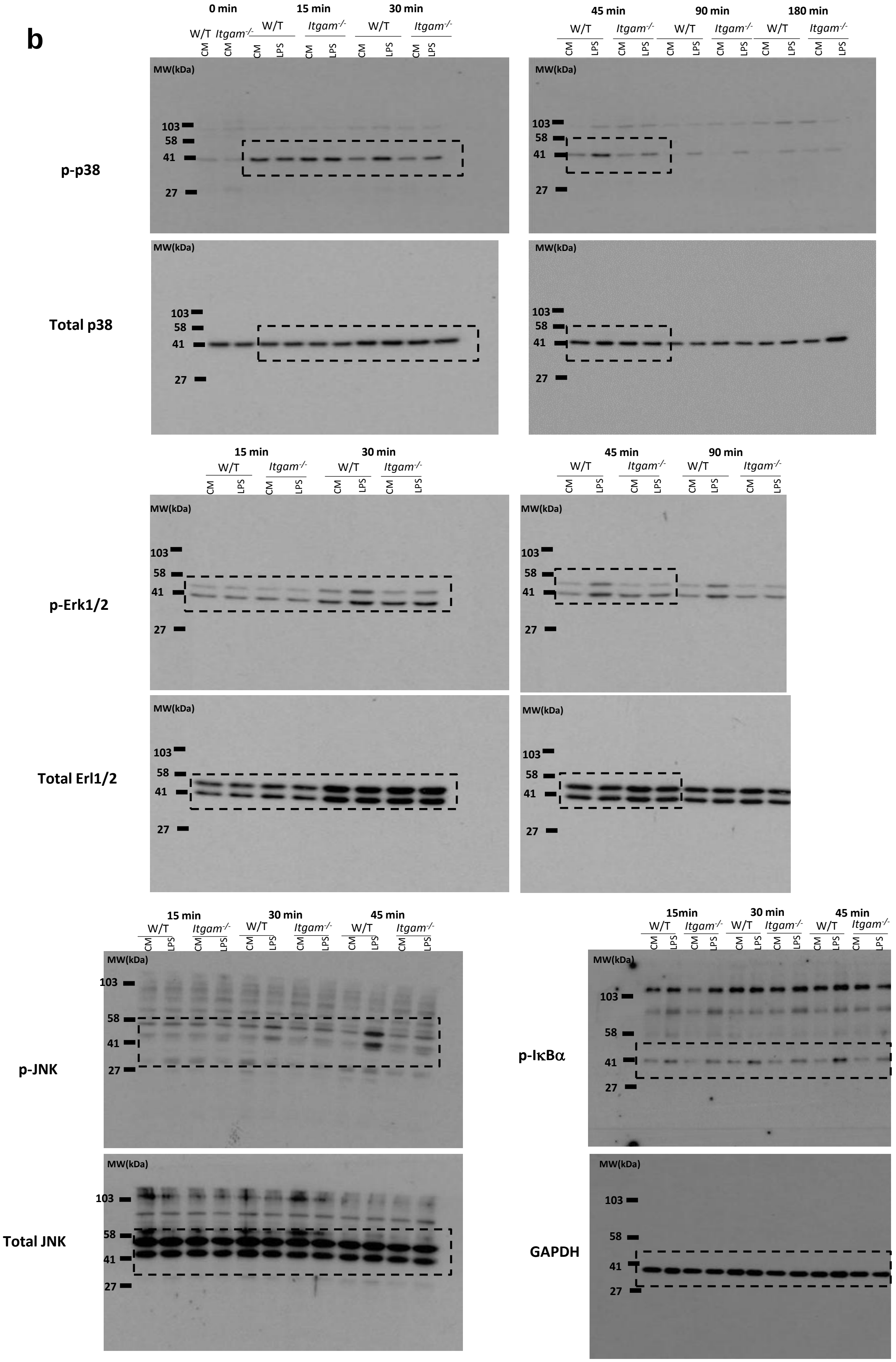

Supplementary Figure S8 : (a) BM-MΦs (b) BM-DCs, full Blots relating to Figure 2.

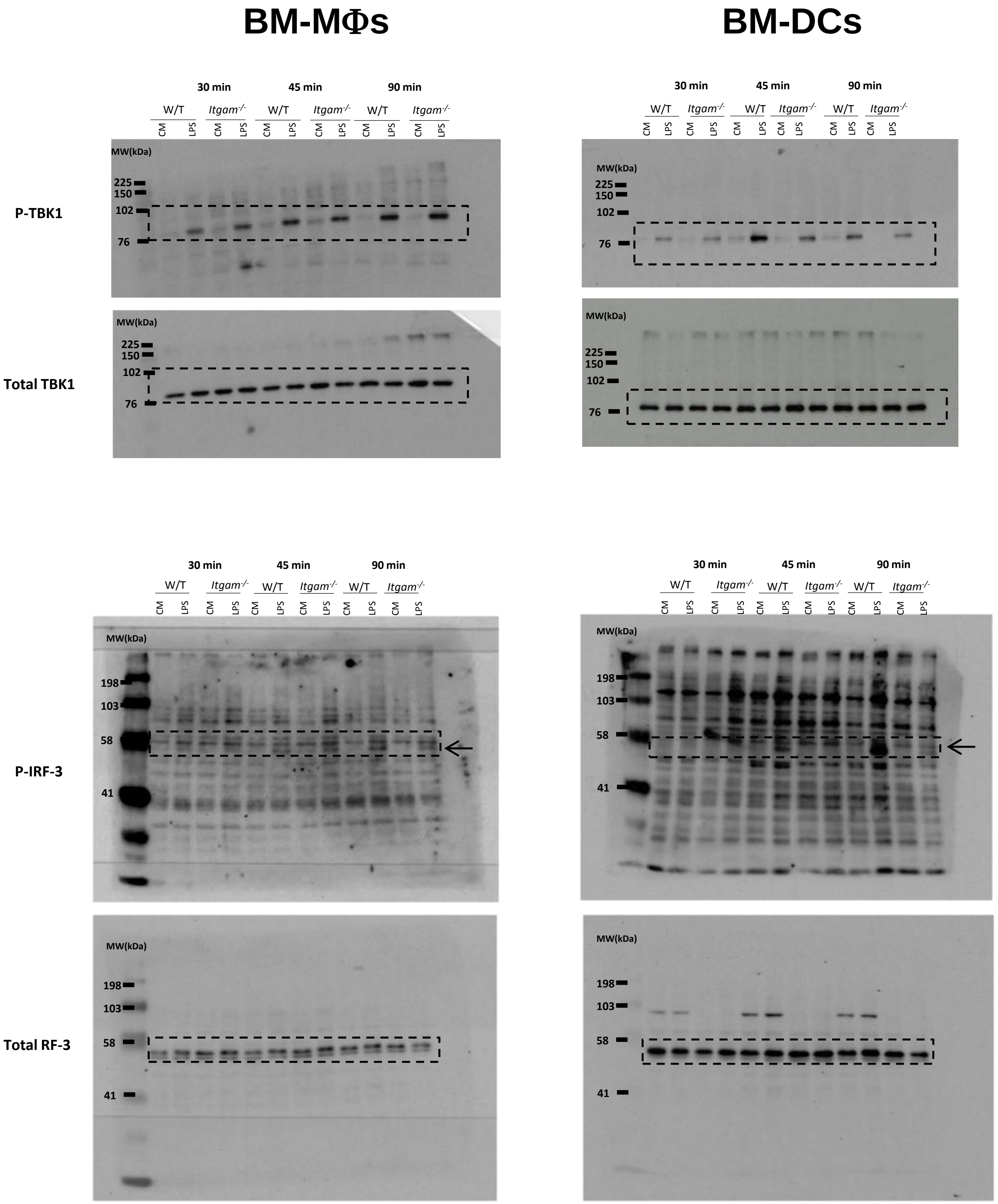

Supplementary Figure S9 : full Blots relating to Figure 5.

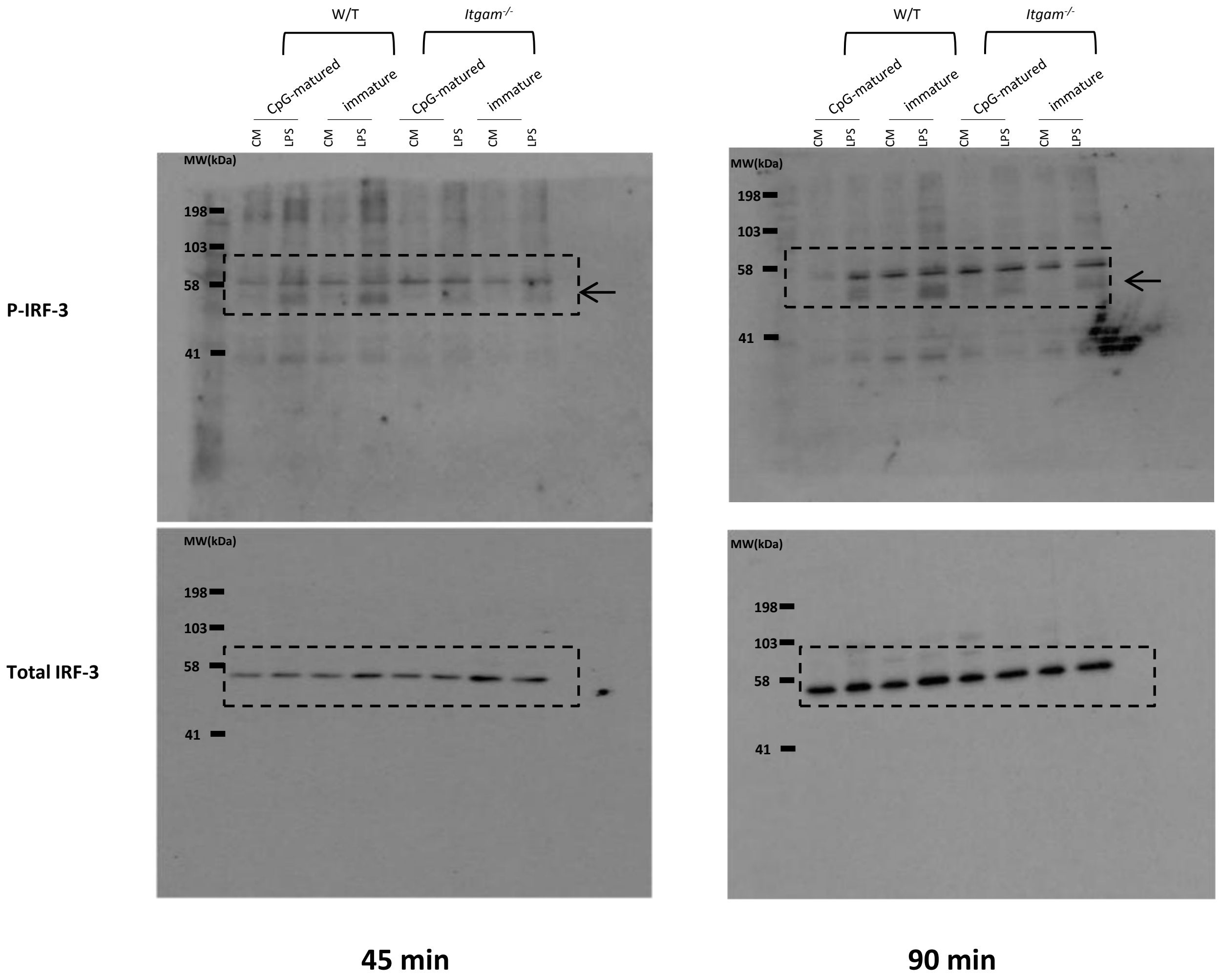

Supplementary Figure S10 : full Blots relating to Figure 6.

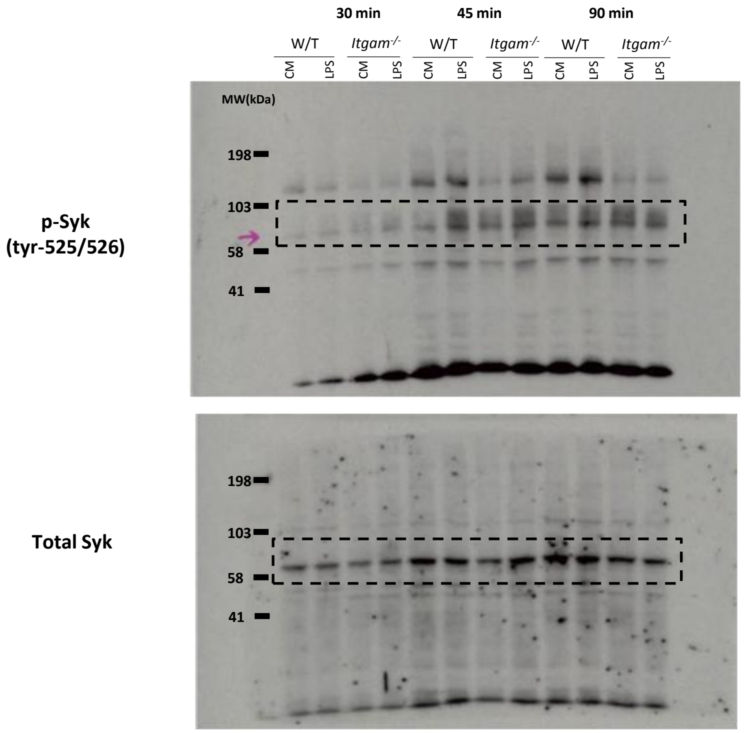

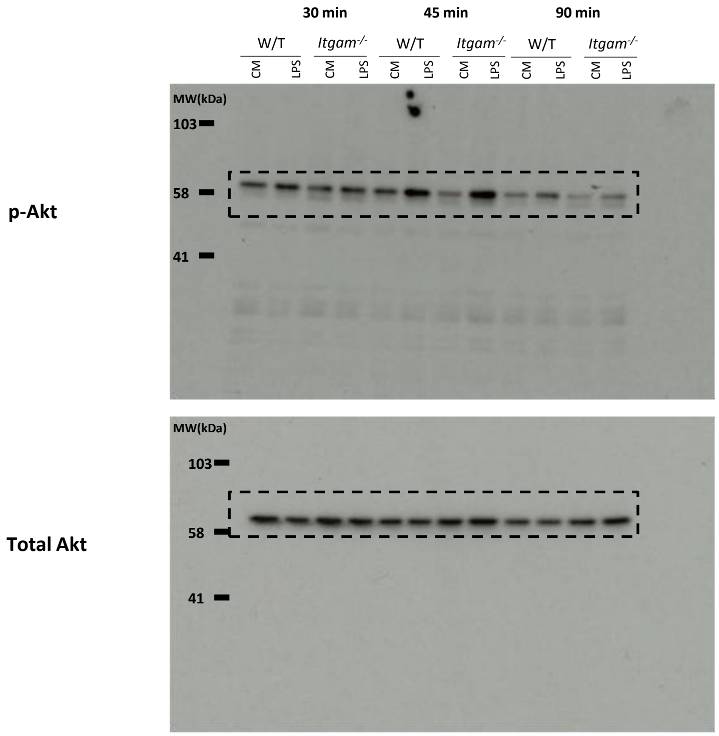

Supplementary Figure S11 : full Blots relating to Supplementary Figure S5.
